# Hidden signatures of early fire at Evron Quarry (1.0 to 0.8 Mya)

**DOI:** 10.1073/pnas.2123439119

**Published:** 2022-06-13

**Authors:** Zane Stepka, Ido Azuri, Liora Kolska Horwitz, Michael Chazan, Filipe Natalio

**Affiliations:** ^a^Kimmel Center for Archaeological Science, Weizmann Institute of Science, 7610001 Rehovot, Israel;; ^b^Bioinformatics Unit, Department of Life Sciences Core Facilities, Weizmann Institute of Science, 7610001 Rehovot, Israel;; ^c^National Natural History Collections, The Hebrew University, 9190401 Jerusalem, Israel;; ^d^Department of Anthropology, University of Toronto, Toronto, ON M5S 2S2, Canada;; ^e^Department of Plant and Environmental Sciences, Weizmann Institute of Science, 7610001 Rehovot, Israel

**Keywords:** Lower Paleolithic, pyrotechnology, spectroscopy, spatiotemporal patterns

## Abstract

Pyrotechnology is a key element of hominin evolution. The identification of fire in early hominin sites relies primarily on an initial visual assessment of artifacts’ physical alterations, resulting in potential underestimation of the prevalence of fire in the archaeological record. Here, we used a suite of spectroscopic techniques to counter the absence of visual signatures for fire and demonstrate the presence of burnt fauna and lithics at the Lower Paleolithic (LP) open-air site of Evron Quarry (Israel), dated between 1.0 and 0.8 Mya and roughly contemporaneous to Gesher Benot Ya’aqov where early pyrotechnology has been documented. We propose reexamining finds from other LP sites lacking visual clues of pyrotechnology to yield a renewed perspective on the origin, evolution, and spatiotemporal dispersal of the relationship between early hominin behavior and fire use.

## Significance

This study reveals the presence of fire in a Lower Paleolithic (LP) site lacking visible signs of pyrotechnology and adds a new LP site to a handful of archaeological sites with evidence associating early hominin–produced artifacts and fire. This research highlights the possibility of extracting “hidden” information on pyrotechnology-related activities from other sites.

Pyrotechnology was a critical element in developing hominin adaptations, society, technology, and biological evolution (1, 2). Evidence associating hominins with fire is rare in sites dating to >500,000 before present (BP) and is limited worldwide to a handful of examples, including Wonderwerk Cave (3) and Swartkrans (both in South Africa) (4), Koobi Fora (5) and possibly also Chesowanja (both in Kenya) (6), Gesher Benot Ya’aqov (Israel) (7, 8), and Cueva Negra (Spain) (9). These sites are within the time range of *Homo erectus*. Subsequent to this date, there is an increase in the frequency and intensity of evidence for burning in archaeological sites, and such evidence becomes widespread after 200,000 BP (10, 11).

Fire identification in archaeological sites primarily relies on visual identification of altered sediments, lithics, and bones (e.g., soil reddening, discoloration, pot lids, warping, cracking, shrinkage, darkening, or calcination). Other complementary analytical techniques extensively used to identify heat exposure of clay sediments, lithics, and bones include magnetic susceptibility (6, 12), Fourier-transform infrared spectroscopy (FTIR) (3, 13–15), thermoluminescence (16–18), and micromorphology (3, 19). Recently, we developed a spectroscopic “thermometer” based on Raman spectroscopy and deep learning (DL) algorithms to estimate the exposure of flint artifacts to heat, independent of visual indications of heat exposure (20).

## Evron Quarry

**Fig. 1.**
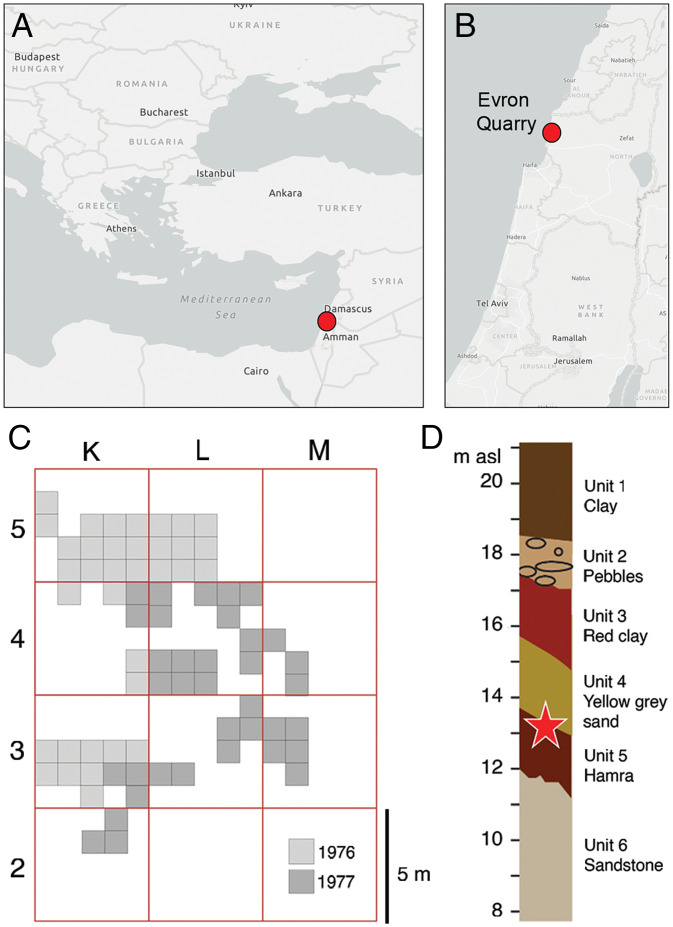
Evron Quarry. (*A* and *B*) The geographic location of Evron Quarry on western Galilee’s coastal plain (Israel). The maps were generated using ArcGIS Pro software. (*C*) Map of 1976–1977 Evron Quarry excavations. The excavated areas are marked in gray hues according to the excavation year. (*D*) Correspondent stratigraphic profile and archaeological layer unit 4 (highlighted by the red star).

The site of Evron Quarry is located on the coastal plain of western Galilee (Israel) (Fig. 1 *A* and *B* and *SI Appendix*, Fig. S1) and is dated by paleomagnetism and optically stimulated luminescence to between 1.0 and 0.8 Mya (21–24). Finds of fossil fauna and Lower Paleolithic tools prompted a series of excavations at this locality by Avraham Ronen in 1976–1977, during which 70 m^2^ of the archaeological layer were excavated (Fig. 1*C*), reaching depths of 14 to 15 m above sea level (25–27). The context of the archaeological findings is an LP occupation horizon (unit 4), described as a yellow-gray sandy layer, with artifacts and fauna found in its lowermost part and at the interface with the underlying red-colored sandy loam (Hamra, unit 5) (25) (Fig. 1*D*).

Our study was conducted on materials from the 1976–1977 excavations (see *SI Appendix* and *SI Appendix*, Fig. S2 for excavation methodology) comprising faunal remains (26), sediments associated with the fauna, and flint tools (25, 28). There is no visual evidence of heat-related features on these materials, i.e., soil reddening, pot lids, discoloration or presence of luster on flint tools, warping, cracking, shrinkage, or color change to faunal remains.

## Results

### Ultraviolet Raman and DL Analysis on Lithics.

**Fig. 2.**
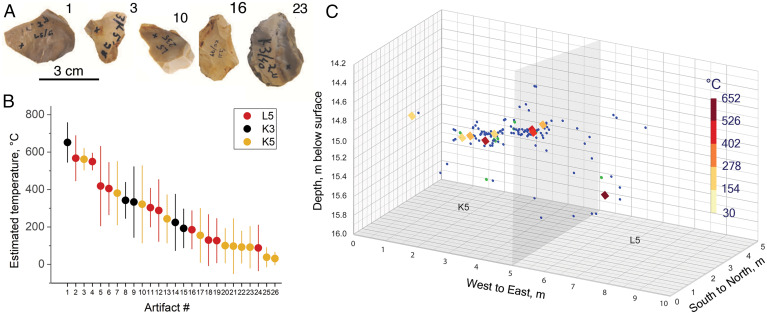
Lithic assemblage. (*A*) Photographs of representative examples of lithic artifacts from Evron Quarry 1976–1977 excavations’ assemblage with no visual features attributed to heat exposure. (*B*) Raman spectroscopy deep learning–based temperature estimation for selected lithic artifacts (*n* = 26) collected from three different squares showing a wide range of temperatures within different squares. (*C*) Spatial distribution of artifacts in the two densest squares, K5 and L5. Diamonds show analyzed lithic artifacts with the color representing estimated exposure temperature, color coded from yellow (lower temperature) to dark orange (higher temperature). Blue dots show lithics not analyzed; green dots, faunal remains.

The lithic assemblage from Evron Quarry unit 4 includes small flakes, retouched flakes, and cores along with a small number of poorly made handaxes as well as small-to-medium-sized cobbles (see discussion of lithic assemblage in *SI Appendix*). The small size of the tools is ascribed to the limitations imposed by the raw material found in the site’s immediate vicinity (25, 28). Analysis of the tool morphology suggests that the pointed tools were not hafted but rather used with force while held in the hand, for tasks, including butchery (28). Squares L5, K5, and K3 have the largest lithic assemblages with provenance data. We chose 26 lithic artifacts that reflect the assemblage’s size range and tool types, including flakes, retouched flakes, and one core (*SI Appendix*, Fig. S3 and Table S1). To estimate the temperature to which the selected Evron Quarry artifacts had been heated, we used ultraviolet (UV) Raman spectroscopy and a DL model (one-dimensional convolutional neural network [1D-CNN]) (*SI Appendix*, Fig. S4) pretrained on modern flint collected from different flint sources throughout Israel and heated to known temperatures under laboratory-controlled conditions (20). This methodology relies on irreversible heat-induced structural modifications that occur to organic and inorganic components of flint while overcoming its intrinsic variability. The advantage of using DL for temperature estimation is that DL models can approximate any nonlinear decision boundaries between heat and the resulting spectral modifications in α-quartz, moganite, and the D and G band spectral regions (20). For this study, we developed an improved DL model (*SI Appendix*, Fig. S4) relative to our previous study (20) with reduced mean absolute error between the true and estimated temperatures from 118 °C to 103 °C and improved Pearson’s correlation coefficient between the true and estimated temperatures from 0.72 (degrees of freedom [d.f.] = 1,567, *P* = 1.8 × 10^−251^, effect size = 0.72, 95% confidence interval [CI] [0.7, 0.74]) to 0.78 (d.f. = 1,567, *P* = 1.98 × 10^−323^, effect size = 0.78, 95% CI [0.76, 0.8]) on the validation experiment (*SI Appendix* and *SI Appendix*, Figs. S5 and S6). Fig. 2*A* shows representative lithics that do not show any visual signs of fire exposure. We used our DL model to estimate the heating exposure temperature from the UV Raman spectra collected from each of the selected lithic artifacts (*n* = 26, *SI Appendix*, Fig. S3). We found that the selected lithics were heated to a wide range of temperatures (Fig. 2*B* and *SI Appendix*, Table S2 and Fig. S7) but were found near each other and at the same elevation within the excavation (Fig. 2*C*). We could not find, in any of the lithics collected from different squares, a cluster of burnt artifacts that fall within a constrained temperature range (*SI Appendix*, Fig. S8). We did not find any compelling correlation between estimated temperatures (heating) of lithics and artifact size (length, thickness, surface area) or type (*SI Appendix*, Fig. S9).

### FTIR Analysis on Fauna and Sediments.

**Fig. 3.**
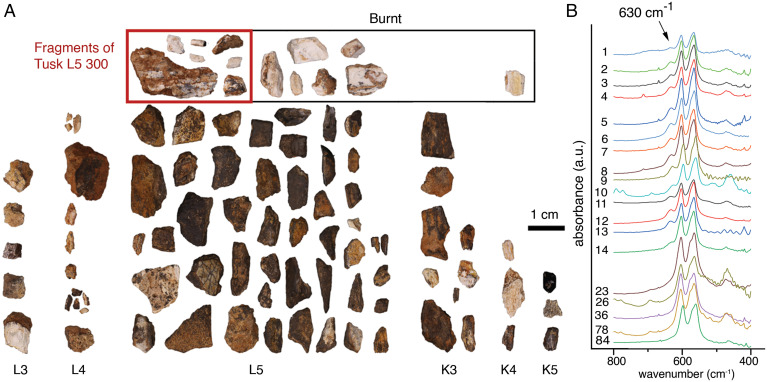
Faunal assemblage. (*A*) Photographic visualization of the whole analyzed faunal assemblage divided by excavation squares labeled as L3, L4, L5, K3, K4, and K5 at the Bottom. The faunal assemblage displays various colors, including dark brown, gray, and white. The red square marks seven analyzed fragments belonging to Tusk L5 300 from square L5, all of which are burnt. The black square marks seven other burnt tusk fragments. Six of them come from square L5 and one from square K4. (*B*) FTIR spectra of all analyzed faunal remains in the spectral region between 800 and 400 cm−1. The topmost 14 spectra (#1 to 14) display the 630 cm−1 hydroxylation peak indicated by the black arrow and diagnostic of exposure to temperatures >600 °C. Five lowermost samples (#23, #26, #36, #78, and #84) represent faunal fragments with no peak at 630 cm−1, indicating that these faunal remains have not been exposed to temperatures >600 °C. The white color of the analyzed faunal fragments is not diagnostic of calcination for this archaeological site.

The faunal assemblage from Evron Quarry unit 4 comprises >250 faunal remains of a range of different-sized herbivores: gazelle, cervids, aurochs, hippopotamus, two species of proboscideans, and a suid (25, 28). They are represented by cranial and postcranial remains, although complete and fragmented teeth predominate, probably due to a preservation bias (see discussion of faunal assemblage in *SI Appendix*). For this study, we used FTIR to analyze a randomly chosen sample of 87 small faunal fragments (<2 cm in length) from different excavation squares, and whose coloration ranged from dark brown, gray, to white (Fig. 3*A* and *SI Appendix*, Figs. S10 and S11 and Tables S3 and S4). We used the presence vs. absence of a peak at 630 cm^−1^ to determine whether the faunal remains had been heated above 600 °C. This peak only appears due to irreversible heat-induced hydroxylation of the bone mineral’s crystal structure after being exposed to 600 °C (29) and cannot be attributed to diagenesis. We identified a prominent 630 cm^−1^ peak in 13 faunal fragments from square L5 and a small peak in a fragment from square K4 (Fig. 3*B* and *SI Appendix*, Fig. S12 and Tables S3 and S4, see discussion in *SI Appendix*, including comparative elemental composition and powder X-ray diffraction (XRD) analysis of bone and tusk in *SI Appendix*, Fig. S13). Scanning electron microscopy (SEM) images confirm that all burnt remains belong to tusk due to the presence of dentinal tubules (*SI Appendix*, Fig. S14). We do not discard the possibility that they belong to the same tusk, given the presence of large fragments of a single proboscidean tusk in square L5 (*SI Appendix*, Figs. S10 and S11 and Table S3). All other 69 faunal remains, comprising fragments of bone as well as tusk, do not display the 630 cm^−1^ hydroxylation peak (Fig. 3*B* and *SI Appendix*, Fig. S10). This includes 19 white-colored faunal fragments, suggesting that color is not diagnostic of heat exposure in this assemblage (*SI Appendix*, Fig. S10).

Several faunal fragments (*n* = 34) had red-colored sediments (Hamra) associated with them, either as lumps of sediment in the bags with fauna or directly adhering to the faunal remains (*SI Appendix*, Fig. S15). We found, using FTIR, that the Hamra predominantly consists of α-quartz and clay, with some sediments displaying minor quantities of calcite. No other phases, such as dahllite, amorphous silica/opal potentially associated with burning, were identified. FTIR analysis of these sediments indicates that all isolated clay fractions show the presence of clay structural water around the 3,600 cm^−1^ region (*SI Appendix*, Fig. S16 and Table S5). These results suggest that the sediments, including those associated with the burnt tusk fragments in square L5, were not exposed to temperatures >400 °C (8) (*SI Appendix*, Fig. S15 and Table S5, see discussion on sediment analysis in *SI Appendix*).

## Discussion

**Fig. 4.**
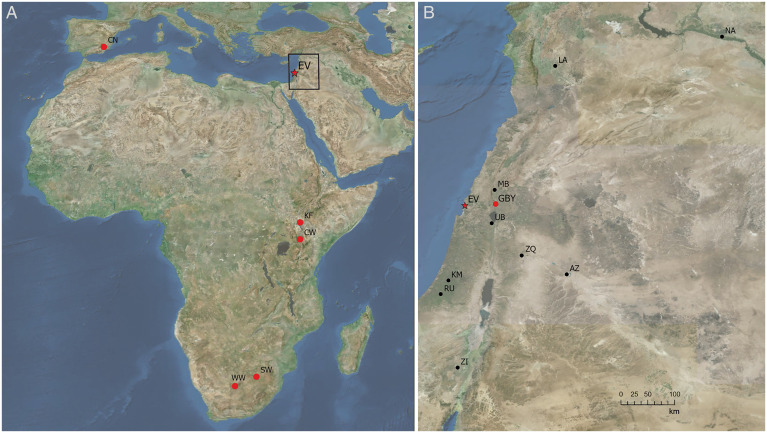
(*A*) Map of the global distribution of Lower Paleolithic sites (> 500,000 y old), including those with evidence associating hominins and fire (red circles) (WW, Wonderwerk Cave; SW, Swartkrans; GBY, Gesher Benot Ya’aqov; CN, Cueva Negra; and KF, Koobi Fora). EQ, Evron Quarry (this study). (*B*) The Levantine geographic location of other roughly contemporaneous Levant sites, including AZ, Azraq; ZA, Zarqa; MB, Ma’ayan Baruch; UB, ‘Ubeidiya; KM, Kefar Menahem; BR, Bitzat Ruhama; ZH, Nahal Zihor; NA, Nadaouiyeh; and LA, Latamne, from which artifacts could be reexamined to potentially expose hidden information on pyrotechnology-related activities. All maps were generated using ArcGIS Pro software.

In this study, we have demonstrated the association of burnt tusk and burnt lithics within a clearly defined archaeological horizon at the LP open-air site of Evron Quarry, adding a new LP site to a handful of archaeological sites with evidence associating early hominin-produced artifacts and fire (Fig. 4*A*). At this stage, we cannot conclusively establish the role of hominins in the presence of fire in the archaeological context at Evron Quarry. The association of artifacts and fauna in such an open-air context might simply reflect the impact of natural fire on the landscape. At the neighboring site of Gesher Benot Ya’aqov (7, 8), spatial patterning in the traces of burning has been interpreted as evidence of hominin intervention since natural fire would be expected to result in homogenous thermal alteration across the burnt area. At the Evron Quarry site, we find differential temperature estimations for lithic artifacts found in the same archaeological horizon (Fig. 2*C*) without any spatial patterning (*SI Appendix*, Fig. S9). Among the faunal remains analyzed, only the tusk is burnt (Fig. 3). It is possible that this patterning is the end result of multiple occupation events. The absence of burnt sediments may indicate secondary reworking of the assemblage such that phantom hearths, such as those found at Gesher Benot Ya’aqov, are not expected to be preserved. We acknowledge that wildfires and patchy vegetation could also result in a heterogeneous distribution of temperatures across the site and that temperature is not a reliable discriminator between wildfires and humanly managed fires (30). Nevertheless, the estimated temperature of the lithic artifacts, the presence of burnt fauna, and their constrained spatial distribution (proximity) within a discrete archaeological horizon, raise the possibility of hominin use of fire.

Our approach highlights the possibility of extracting “hidden” information on pyrotechnology-related activities from artifacts beyond the classical visual initial assessment. Thus, we propose reexamining artifacts unearthed from other LP sites, including those located in the Levant (Fig. 4*B*) may potentially broaden our spatiotemporal understanding of the relationship between early hominins and fire and its potential implications for transmission (3) and dispersal paths (11) of fire-related knowledge at the local and global scale.

## Materials and Methods

### Sample Collection.

All materials analyzed for this study derived from collections excavated by Avraham Ronen (University of Haifa) at Evron Quarry in 1976 and 1977 (25–27). The original quarry was subsequently infilled, but during excavations in 1985, a pit was dug ∼10 m to the south of the previous quarry excavation, and the archaeological horizon was reached here at 9.4 to 8.6 m above sea level, a drop of ∼5 m from the previously excavated locality, probably reflecting the downward slope toward a paleo-river channel (25). Material from the 1985 season was not included in our study as the lithics from this excavation have been mislaid. Although during the 1976–1977 season, the excavators [Prausnitz and Ronen (27) and Ronen (25)] noted the presence of at least two levels of different living floors, the available data for the elevation of the finds indicate only a single occupation horizon in squares K5 and L5, the squares that provided the bulk of the sample used for the present study. However, there are two horizons evident in finds from square L3 that were not sampled for this study.

The 1976–1977 excavations employed a grid marked in 5 × 5 m units designated by capital letters and numbers (e.g., L4, L5). These were subdivided into 1 × 1 m subunits denoted by small letters (e.g., L5a, L5b). *SI Appendix*, Fig. S2 illustrates the layout of the excavation and shows the parts of each square that were excavated. In no case was the complete 5 × 5 m square excavated. Excavation notes state that sieving was regularly employed. Although mesh size is not specified, it was probably in the 5-mm range, given the small size of the lithics and faunal fragments recovered. Most lithic objects identified during the excavation were plotted with three coordinates, although some were collected and documented by square only, i.e., deriving from within the entire 5 × 5 m area. For larger faunal remains, some were plotted using three coordinates, and for a few, their 1 × 1 m subsquare numbers were also listed. However, most faunal remains were attributed only to their 5 × 5 m squares. Plan drawings were also made showing find locations, and provenience information was written in ink directly on most artifacts. Following excavation, the lithic finds were curated at the University of Haifa, while the fauna was curated at the National Natural History Collections of the Hebrew University. The archaeological material is currently on loan from Haifa University to L.K.H. (The Hebrew University). In 2022, all artifacts will be passed on to the Israel Antiquities Authority for permanent curation.

All maps were created in ArcGIS Pro. Basemap is licensed under the Esri master license agreement.

### SEM.

Samples were attached with carbon tape to aluminum T-stubs (1-cm diameter). Samples were imaged with a backscattered electron detector at an accelerating voltage of 15 kV using low vacuum Phenom ProX (Thermo Fisher Scientific). Elemental X-Ray analysis was performed under the same conditions (15 kV) and collected for 60 s.

### FTIR Analysis.

A few milligrams of sediment sample (*n* = 34) and faunal fragments (*n* = 87) were powdered using an agar mortar and pestle and mixed with 5 mg KBr. The mixture was pressed into a 7-mm die using a Pike hand press and analyzed with a Thermo Nicolet iS5 FTIR spectrometer. FTIR spectra were collected by performing 32 scans at a resolution of 4 cm^−1^ in the 4,000- to 400-cm^−1^ spectral range. The FTIR spectra were acquired and baselined using Omnic software and replotted using OriginLab Pro-2018 (b9.5.0193).

### Sediment Samples.

For samples in which α-quartz peak (approximately 1,084 to 1,086 cm^−1^) shadowed clay peak (approximately 1,032 to 1,035 cm^−1^), we separated the clay-rich sediment fraction using density separation (31). First, 600 µL 1N HCl was added to ∼50 mg of sediment, allowing it to react at room temperature for 5 min. Then, the samples were centrifuged (5,000 rpm for 5 min at room temperature), and the supernatant was removed. Next, we added 300 to 400 µL of sodium polytungstate solution (2.5 g/mL, ∼85%; Sigma) to the acid-insoluble pallet, vortexed, sonicated for 10 min at room temperature using an ultrasonic bath (MRC Laboratory), vortexed, and centrifuged (5,000 rpm for 5 min at room temperature). Then, the supernatant containing the clay-rich fraction was removed, washed with deionized water, vortexed, and centrifuged (5,000 rpm for 5 min at room temperature) three times, and the water was removed. Finally, the pellets were dried in an oven at 40 °C and reanalyzed with FTIR.

### Bone Samples.

Identification of the peak at 630 cm^−1^ attributed to hydroxylation of the bone mineral after exposure to heat above 600 °C (29, 32) was carried out using OriginLab Pro-2018 (b9.5.0193).

### UV Raman Spectroscopy of Flint-Stone Tools from Unit 4.

Selected flint lithic artifacts (*n* = 26) were analyzed by Raman microspectroscopy (laser 325 nm). The mapping was collected by a Horiba LabRAM HR Evolution spectrometer (20). The system has an 800-mm focal length spectrograph with interchangeable gratings mounted with an open-electrode, front-illuminated, cooled charge-coupled device detector. The sample is placed under a modular microscope (Olympus BX-FM) using a 40× objective. An automated XYZ stage was used to move the sample under the objective to map a sample section. Twenty-seven spectra were recorded for each sample using the following conditions: 325 nm (UV), grating 1,800 (500 nm), Syncerity Open Electrode detector, hole 100, and Intensity Correction System correction.

### Temperature Estimation of Lithic Artifacts Using Deep Learning Models.

Temperatures were estimated using a 1D-CNN and a fully connected artificial neural network (FC-ANN), as described elsewhere. When compared to FC-ANN (20), the 1D-CNN model reduces the mean absolute error (MAE) between the true and estimated temperatures on the validation experiment from 118 to 103 °C, as well as the MAE between the true and averaged estimated temperatures from 66 to 55 °C (*SI Appendix*, Figs. S5 and S6). See *SI Appendix* *Deep Learning* *Approach to Evron Quarry lithic assemblage* – *Model Selection* for details on the model.

## Supplementary Material

Supplementary File

## Data Availability

The archaeological material is currently on loan from Haifa University to L.K.H. In 2022, all artifacts will be passed on to the Israel Antiquities Authority for permanent curation. The complete dataset and DL code are available at the online public repository: https://github.com/fnatalio/Evron_Quarry. All other study data are included in the article and/or *SI Appendix*.
