# Supplementary material for: Hidden signatures of early fire at Evron Quarry (1.0 to 0.8 Mya)
Source: Proc Natl Acad Sci U S A. 2022 Jun 13;119(25):e2123439119. doi: 10.1073/pnas.2123439119 (PMC9231470; doi:10.1073/pnas.2123439119)
Supplement: Supplementary File [file pnas.2123439119.sapp.pdf]

Supporting Information for

Hidden signatures of early fire at Evron Quarry (1.0 – 0.8 Mya)

Zane Stepka[1#], Ido Azuri[2#], Liora Kolska Horwitz[3], Michael Chazan[4], Filipe Natalio[1,5*]

[1] Kimmel Center for Archaeological Science, Weizmann Institute of Science, 7610001, Rehovot, Israel

[2] Bioinformatics Unit, Department of Life Sciences Core Facilities, Weizmann Institute of Science, 7610001, Rehovot, Israel

[3] National Natural History Collections, The Hebrew University, 9190401, Jerusalem, Israel

[4] Department of Anthropology, University of Toronto, 19 Ursula Franklin Street, Toronto, Ontario M5S 2S2, Canada

[5] Department of Plant and Environmental Sciences, Weizmann Institute of Science, 7610001, Rehovot, Israel

[#] shared contribution

* corresponding author: Filipe Natalio, Nella and Leon Benoziyo Building for Biological Sciences, Room 556B, Weizmann Institute of Science, 234 Herzl Street, Rehovot, 7610001 Israel, +972 8 934 6101, filipe.natalio@weizmann.ac.il

This PDF file includes:

Supplementary Text

Figs. S1 to S16

Tables S1 to S5

References 1 to 30

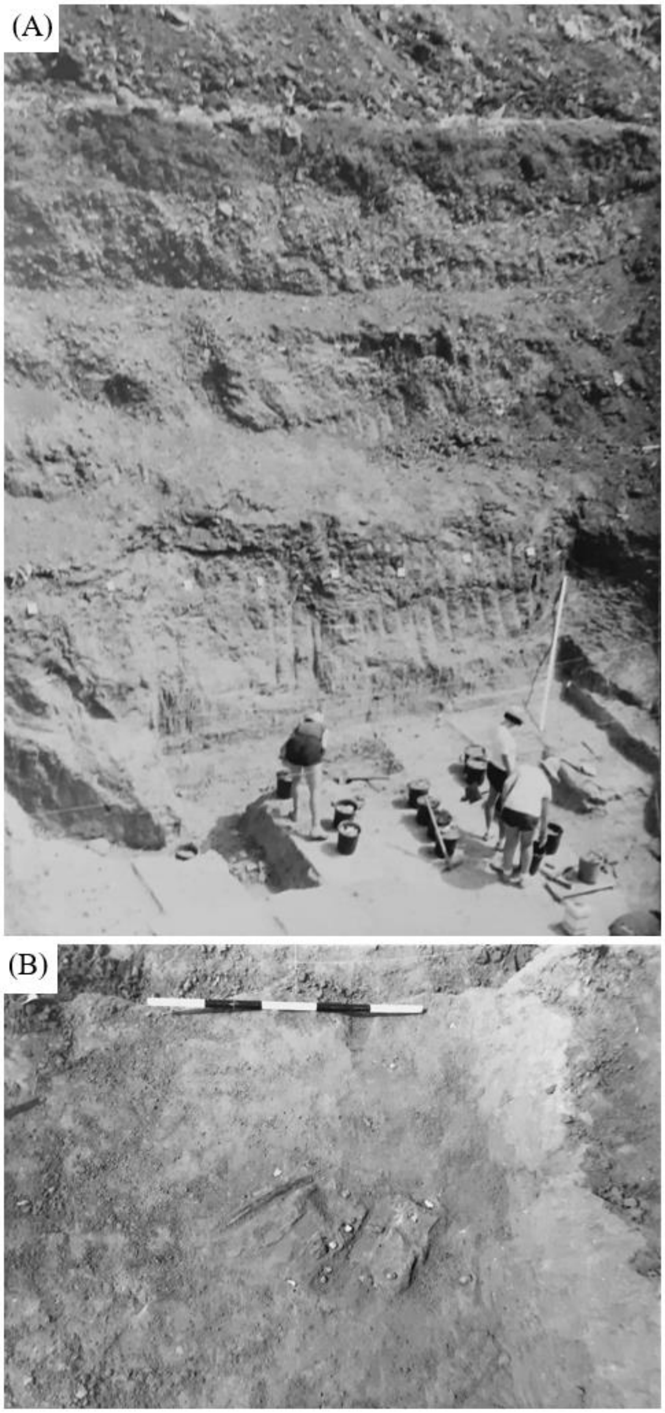

Fig. S1. (A): Overview of 1976-1977 excavations at Evron Quarry showing the depth of the archaeological deposit. (B): Detail of 1976-1977 excavation at Evron Quarry showing association of lithics and fauna. Images from the Evron Quarry excavation archive.

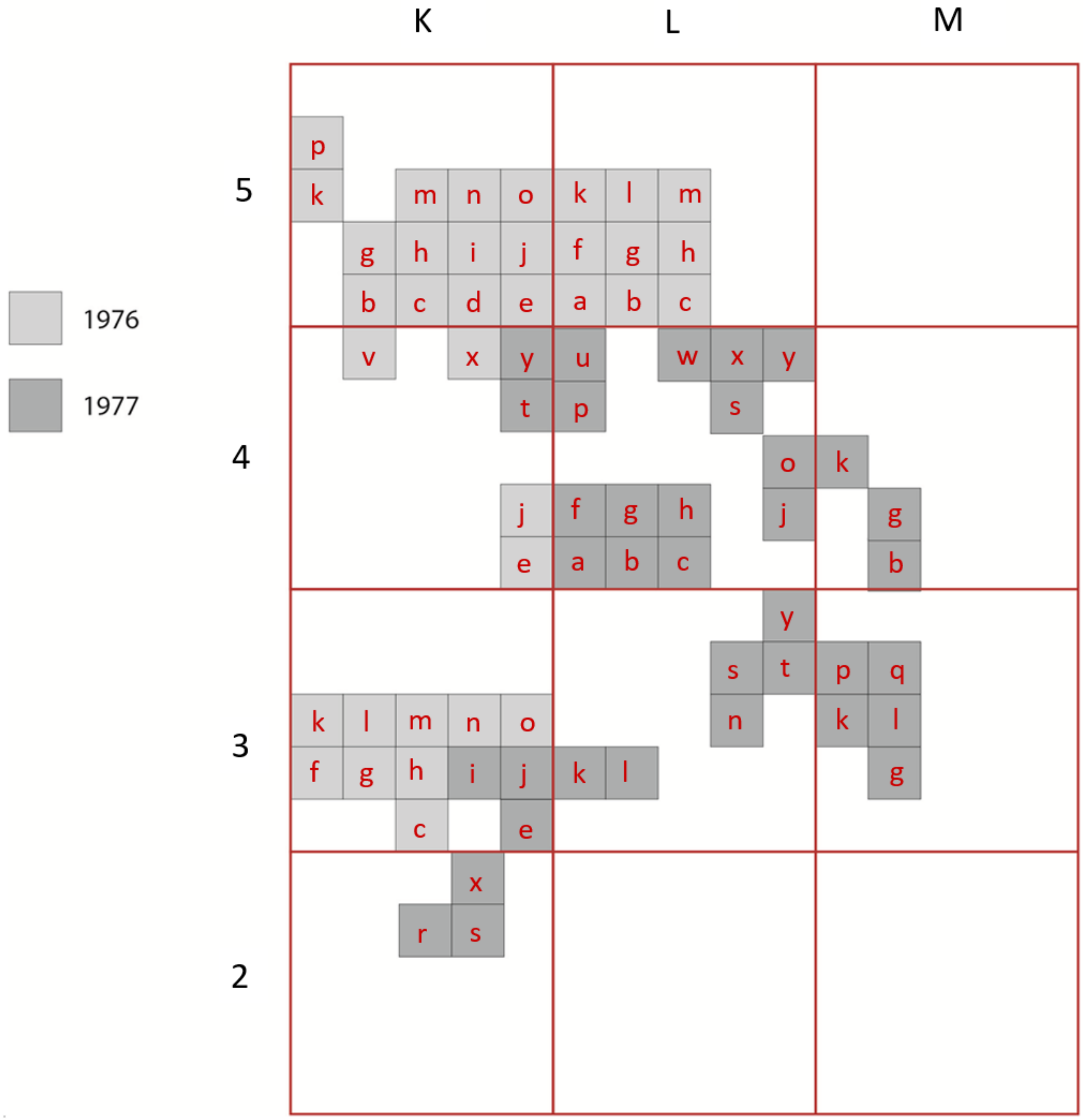

Fig. S2. Grid of the 1976-77 excavations at Evron Quarry. Red-bordered squares (e.g. K5, L5) are 5 x 5 m large. The sub-squares (with grey borders) are 1 x 1 m large. Different grey shades show the sub-squares excavated in different years.

The Evron Quarry lithic assemblage

The lithic assemblage from Evron Quarry was described first by Ronen (1, 2) and subsequently by Chazan (3). The Quarry assemblage is clearly distinct from the later Acheulean industries found near the surface at the neighboring locality of Evron Zinat, characterized by large numbers of well-made handaxes (4). The Evron Quarry industry is dominated by small flake production, including cores, unretouched, and retouched flakes. In addition, there are a small number of larger bifaces associated with this context, all of which are poorly made on irregular raw material (3*: Figure 7*). Ronen (2) pointed to the role of raw material in determining the character of this industry, which relies on the exploitation of river cobbles available in the site's immediate vicinity, most of which are of small size. Ronen (2) recognized the presence of pointed retouched flakes in the assemblage and suggested that these might have been hafted for use as spears. Chazan (3) confirmed Ronen's typological observations and found a frequent association of impact fractures with retouched points. Points were formed by combining deep Clactonian notches on one edge, paired with convex retouch on the opposing edge. However, Chazan's analysis (3) found that the retouched points' morphology was not well suited for hafting, with some pieces having multiple pointed elements formed by retouching. Following Ronen (2), Chazan suggests that the Evron lithic technology reflects the constraints of the locally available raw material and points to a lack of long distance transport of raw material. In contrast to Ronen, Chazan interprets the retouched points as handheld tools and the impact fractures as evidence that these tools were used with great force, likely in the process of butchery. Small tool industries are also found in other Lower Paleolithic contexts on the Coastal Plain of Israel, such as Kefar Menahem Halulim (4) and Bitzat Ruhama (5).

The 26 lithic artifacts analyzed for this study are a representative subset of the sample from the Ronen 1976-77 excavations at Evron Quarry that was available for study from the University of Haifa. The analyzed assemblage is shown in Fig. S3 and described in Table S1. The analyzed assemblage includes cores, flakes, and retouched flakes—including four points.

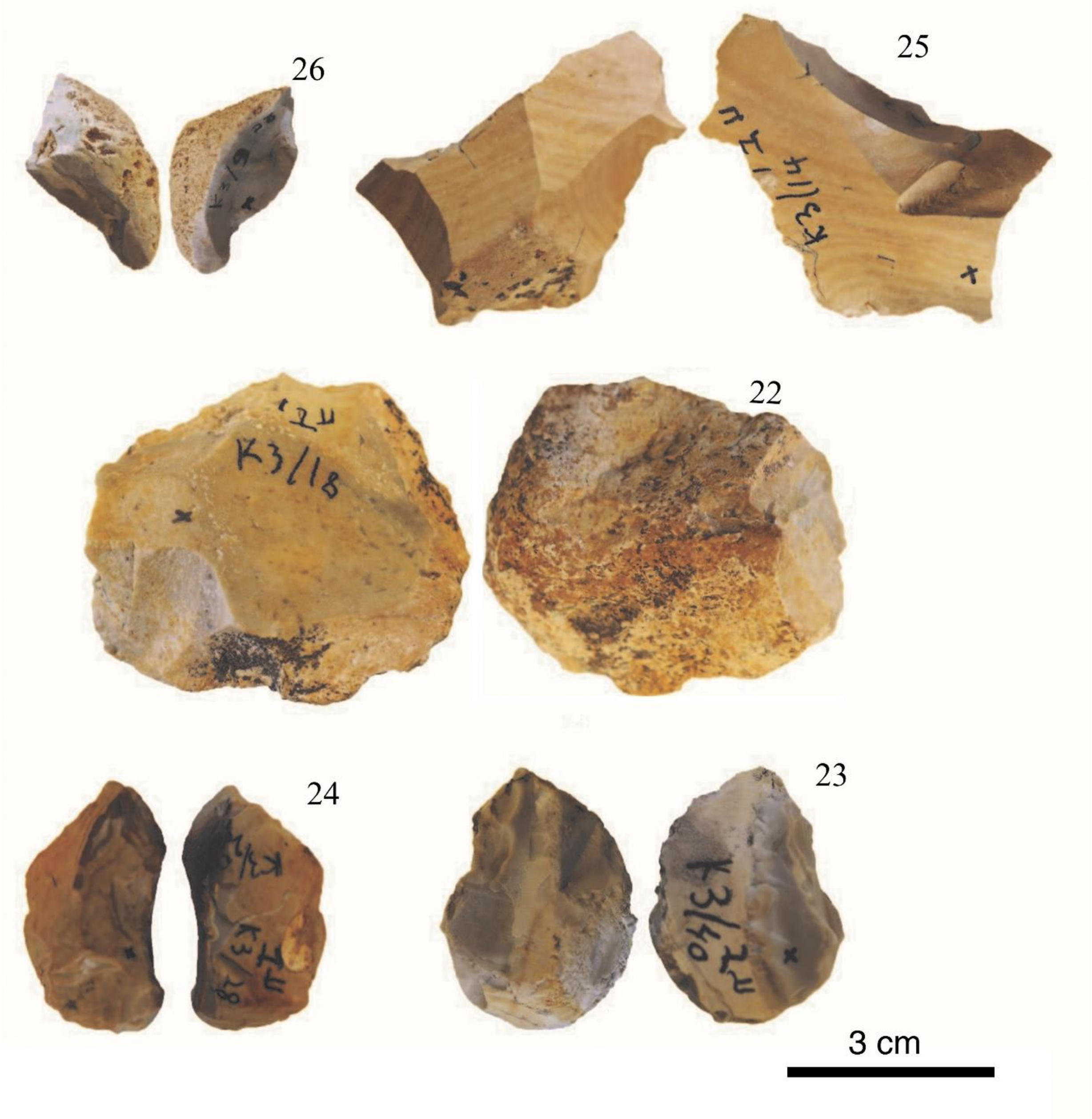

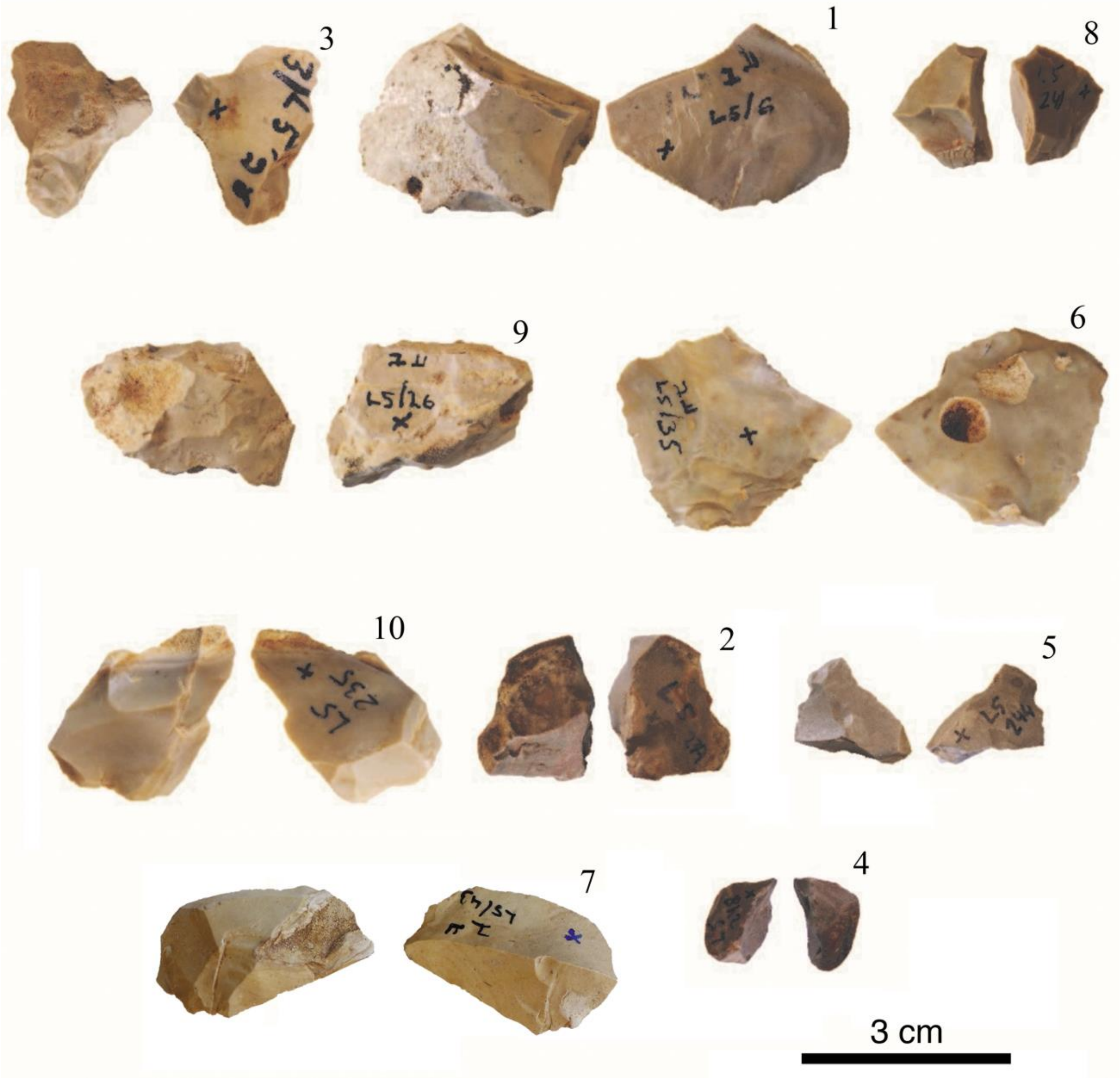
3
1
8
9
6
10
2
5
7
4
3 cm

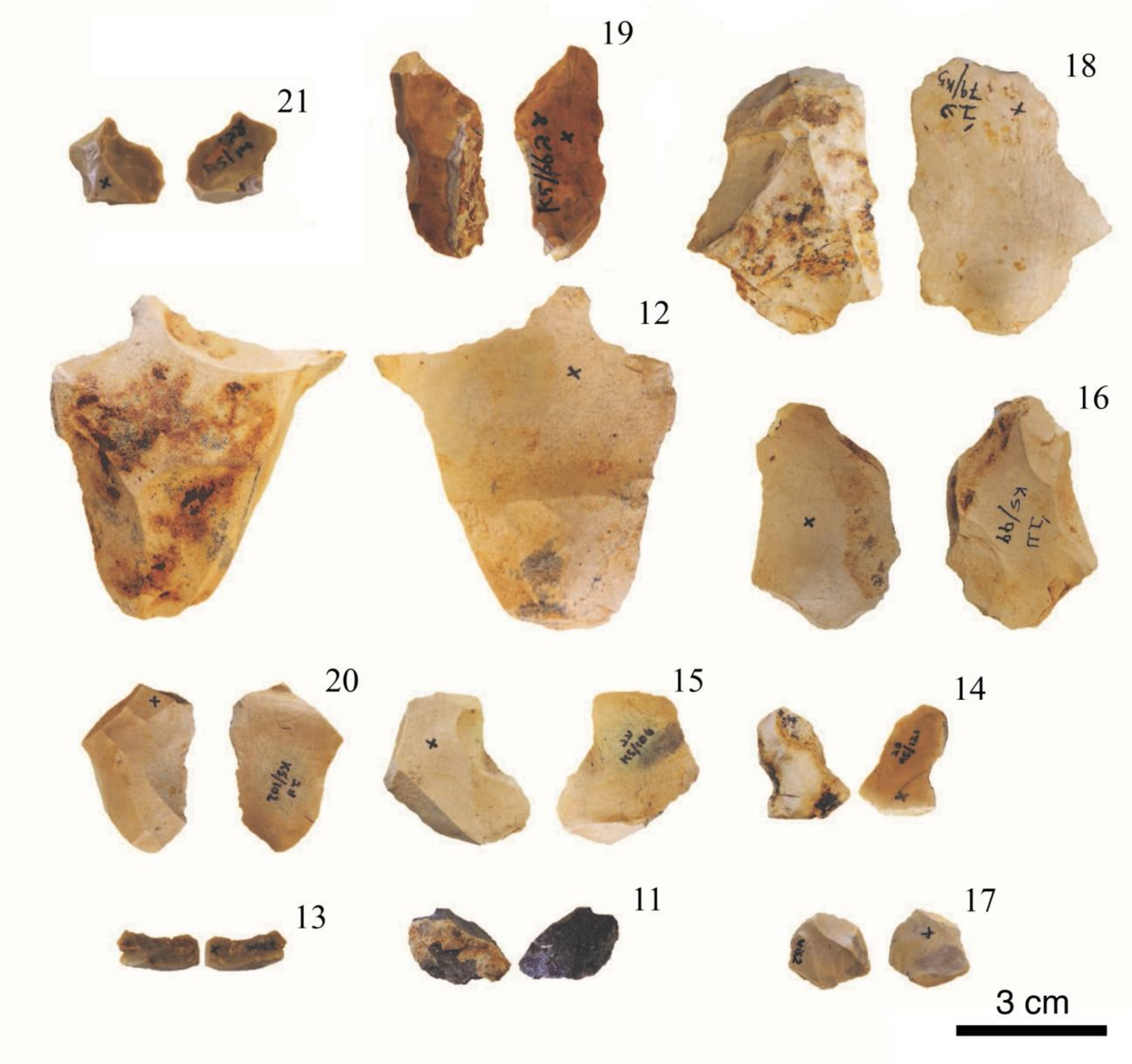

Fig. S3. Photographic images of twenty-six lithics from squares L5, K5, and K3 measured by UV Raman spectroscopy. All these artifacts come from Evron Quarry archaeological site 1976-77 excavations. None of the analyzed lithic artifacts (including the entire collection previously analyzed by Chazan and reported elsewhere (3)) show visible evidence of exposure to heat, such as a change in coloration, luster, or presence of concave depressions (pot lids). Lithic numbering corresponds to numbering in Table S1 (descriptions) and Fig. S2 (temperature estimation results).

Table S1. Description of the lithic artifacts.

#	Flint artifact	Length (mm)	Width (mm)	Thickness (mm)	Nature	Cortex
1	L5_6	32.4	27.9	12	FLAKE FRAGMENT	Y
2	L5_239	20.0	13.7	11.4	FLAKE	N
3	L5_3	25.8	20.8	9	RETOUCHED POINTED	Y
4	L5_248	14.4	8.5	7.7	FLAKE	Y
5	L5_244	17.6	13.5	6.2	FLAKE	N
6	L5_35	34.4	28.1	5	COMPLETE FLAKE	N
7	L5_43	27.7	17.1	10.8	SPLIT FLAKE?	-
8	L5_241	17.8	13.9	8.8	FLAKE	N
9	L5_26	30.9	17.7	13.2	INDETERMINATE FLAKE	Y
10	L5_235	31.1	19.1	9.5	FLAKE	N
11	K5_waste	30	12	7.1	FLAKE	Y
12	K5_84	64.7	59.9	15.9	RETOUCHED POINTED	Y
13	K5_wis1	16.4	6.5	4.7	FLAKE	N
14	K5_121	24.2	17.4	7.5	NOTCHED FLAKE	Y
15	K5_106	33.1	24.9	8.4	NOTCHED FLAKE	N
16	K5_99	48.7	31.6	7	FLAKE (UTILIZED)	N
17	K5_wis2	17.2	14.9	4.4	FLAKE	N
18	K5_79	55	40.2	9.9	FLAKE (RETOUCH)	N
19	K5_66	48	18.2	8.9	NOTCHED FLAKE	Y

20	K5_102	34.3	23.5	7.8	FLAKE (IMPACT?)	N
21	K5_14	20.9	16.9	7.7	NOTCHED FLAKE	N
22	K3_18	54.2	46.8	25.2	CORE	Y
23	K3_40	38.5	25.5	18.7	RETOUCHED POINTED	Y
24	K3_28	36.9	23.8	16.2	INDETERMINATE FLAKE	N
25	K3_14	48	35	22	RETOUCHED POINTED	N
26	K3_9	32	18.1	13.4	INDETERMINATE FLAKE	Y

Deep Learning Approach to Evron Quarry lithic assemblage

We applied deep learning (6, 7) to learn patterns in Raman spectra of flint artifacts that are correlated with temperatures to which flint has been heated. The method has two main parts – first, developing, training, and validating a deep learning model on reference flint samples that have been heated to known temperatures experimentally in a laboratory and, second, applying the trained model to unknown samples (here – lithics from Evron Quarry). Since we have a reference collection of oven-heated flint samples from different localities in Israel, we employed the supervised deep learning approach to correlate Raman spectra with temperatures to which flint had been heated. We used the same reference flint samples and validation experiment to train and validate a deep learning model as described elsewhere (8). The reference samples are flint nodules collected from ten different sources across Israel and heated to different temperatures (see (8) for details on the samples and methodology). For this study, we developed, trained, validated, and applied a new one-dimensional convolutional neural network (1D-CNN) model, which performs better than the fully connected neural network (FC-ANN) deep learning model used in (8). We applied both models to the Evron Quarry lithic assemblage for comparison purposes, and we report the results estimated by both DL models.

Input and output to the deep learning model

The input to the deep learning model are Raman spectra intensities of each spectrum in three wavenumber regions of Raman spectra (see below), and the output (also referred to as the target) is the temperature that the geological flint was heated (artificially). The preprocessing steps are the same as applied in (8). However, while in (8), intensities for all range of wavenumbers were used, here we selected only those regions that correspond to wavenumbers between 102-313 cm^{-1}

(overlap tones of α-quartz and moganite bands), 400-567 cm^{-1} (α-quartz and moganite bands), and 1163-1798 cm^{-1} (D and G bands), that contain most of the spectral information (9), and suppress noise.

Model selection

We applied the one-dimensional convolutional neural networks (1D-CNN) approach. The 1D-CNN model is a more advanced deep learning model than the fully connected neural network (FC-ANN) model applied before for temperature estimation of lithic artifacts (8). The 1D-CNN model is from the CNN deep learning models family. In this approach, each Raman spectrum is a 1D vector that contains the Raman intensities that serve as input to the model. These intensities are convolved with 1D filters, where each filter is a vector of weights that extracts the most informative intensities that are correlated with the target. The best filter weights that extract the most important informative intensities are learned by the model via the optimization process of mapping between the input and the target. Then, a non-linear activation function (here, we used Rectified Linear Unit function 'ReLU') is applied on the convolution output, which enables the model to learn complex and non-linear relations with the target.

After that, a pooling operation is applied (here, we applied 'MaxPooling'). The pooling operation reduces the dimensions of its input while keeping the most important information. The convolution, activation, and pooling operations refer to layers in the model and form a block of operations. More blocks enable to learn higher-level features and more complex patterns that are correlated with the target. Each subsequent block has an equal or higher number of filters than the former block, while the pooling operation reduces the sample dimensions. These blocks extract the predictive features. These features are flattened to one vector and fed to fully connected layers,

followed by a non-linear activation function to yield the target. The fully connected layers learn further complex patterns from the features fed to them. The process of Raman spectrum's processing through the model's architecture is depicted in Fig. S4.

Ten different architectures were examined during the development of the DL model's architecture. The architectures differ by the number of blocks, filter sizes, number of fully connected layers, and number of neurons in the fully connected layers. For each architecture, we applied 10-fold cross-validation splitting between the training set and testing set, where each training fold contains data from nine sites and the tenth site is used for testing (our data set contains geological flints from 10 different sites). The model was trained on the training set for 80 epochs with a batch size of 24, with the Adam optimizer and learning rate of 0.001. Then, temperature estimation was done on the testing set. The closer the estimated temperatures are to the true ones, the smaller the error and the better the model estimates the temperatures that flint was heated. We averaged the performances on the 10-fold testing and chose the model which yielded the optimal average performance. The architecture of the chosen model is shown in Fig. S4. We note that slightly different results can be obtained for different runs during neural network training due to randomness-related processes of the training procedure and the random initialization of its parameters. We experimented with the training process for the different architectures a few times. We chose architectures that yielded performances that are on par with or better than the FC-ANN. Eventually, the 1D-CNN model performed the best and yielded results with the mean absolute error (MAE) reduced by several degrees Celsius (see below Validation experiment results section) compared to the FC-ANN on some splits of the 10-fold cross-validation experiment and other testing splits. After the model architecture tuning, we trained the selected 1D-CNN model on all

the folds together, saved it to a disc, evaluated its performance with a validation experiment, and employed the model to estimate temperatures to which flints from the Evron Quarry had been heated. We compared temperature estimation results yielded by the previously used FC-ANN model (8) and 1D-CNN model. While the comparison demonstrates a better performance of the new 1D-CNN model, we note that both models yield comparable results. Since the two models derived from two different families of deep learning models, the fact that they both succeed in estimating temperatures to which flint has been heated increases our confidence in the results by any one model.

1D CNN Model

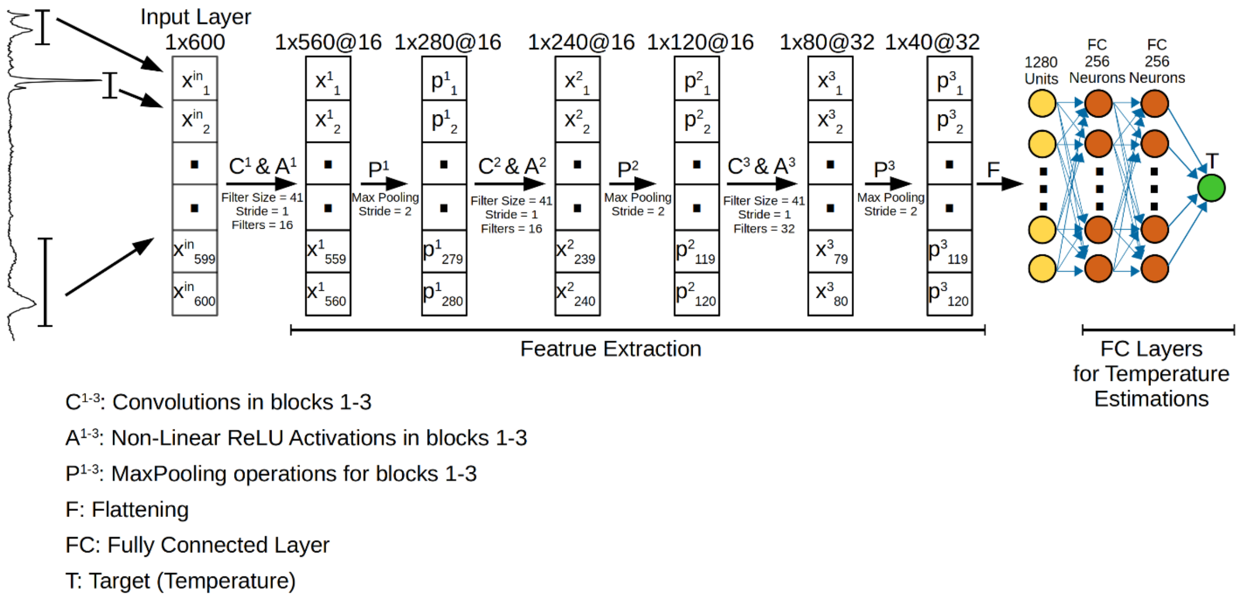

Fig. S4. 1D-CNN model architecture used to estimate the temperature to which flint artifacts had been heated. A Raman spectrum is shown on the left-hand side, with line segments marking the spectrum areas selected as input to the model (*Input Layer*). The model is composed of convolutional layers, non-linear ReLU activations, and MaxPooling. These layers form blocks that extract features from the Raman spectra that are correlated with the target (temperatures). The box blocks and their annotations illustrate the data dimensions after it has been passed through each block/layer of the model (e.g., 1x560@16 denotes that this layer transforms the input (one spectrum of 600 Raman intensity values) to 16 most informative features of size 560 each). After the last block, the features are flattened and fed into Fully Connected Layers for the flint temperature estimation.

Validation experiment

To validate the model's performance in estimating temperatures to which flint had been exposed, we tested the model on a reference collection of geological flint samples from different sources in Israel that were heated in an oven (8). The flint samples used for the validation experiment were not used during any of the earlier stages of training and tuning the model. The validation experiment was conducted on ten flint fragments for each temperature, with each flint fragment deriving from a different geological source in Israel. We used seven temperature categories and a total of 70 flint fragments for this experiment. These samples come from the same collection as the samples used for training, but the particular fragments used for the validation experiment were by no means used for training and tuning the model. Furthermore, Raman measurements on the validation samples were conducted independently, several weeks after the measurements of the samples that were used for training. This randomization ensures minimizing bias from our estimations.

The temperature estimations are shown as histograms in Fig. S5 and box plots in Fig. S6. The MAE was reduced from 118 °C with FC-ANN to 103 °C with 1D-CNN. The average MAE ('average' is for averaging the temperature estimations for each category and calculating the MAE between the average values and true categories values) was reduced from 66 °C with FC-ANN to 55 °C with 1D-CNN. In addition, the Pearson correlation coefficient between the true and estimated temperatures increased from 0.72 for the FC-ANN (d.f. = 1567, $P = 1.8 \times 10^{-251}$, effect size = 0.72, 95% CI [0.7, 0.74]) to 0.78 for the 1D-CNN (d.f. = 1567, $P = 1.98 \times 10^{-323}$, effect size = 0.78, 95% CI [0.76, 0.8]) on the validation experiment.

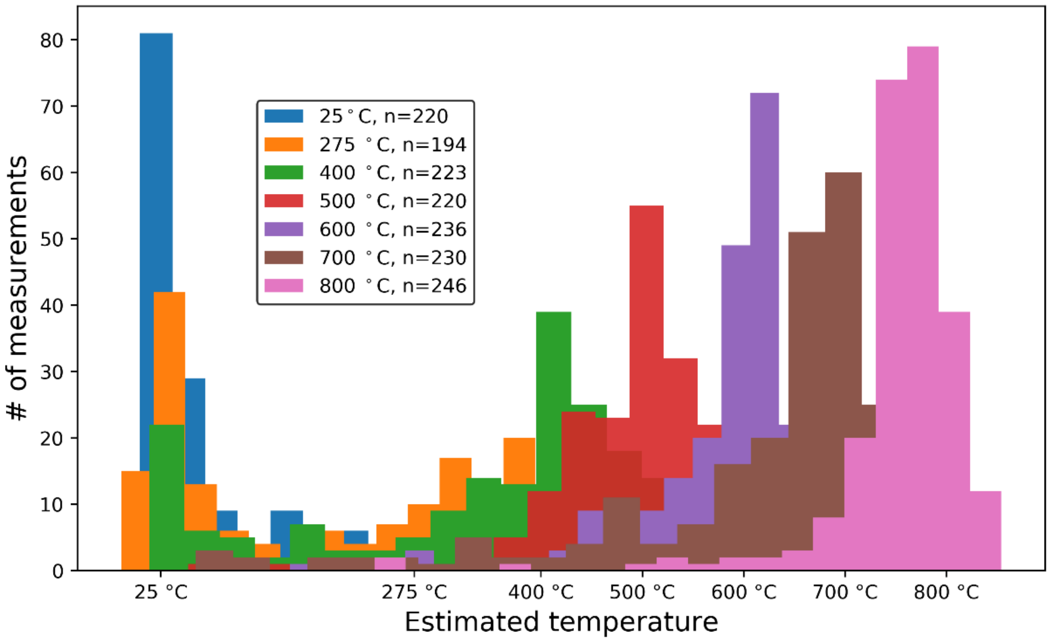

Fig. S5. Histograms of the temperature estimations for the validation experiment of the 1D-CNN model for each temperature category. Ten flint fragments were used for each temperature category (each from a different geological source). n in the figure's legend represents the number of individual measurements (numerous measurements were taken from each flint fragment).

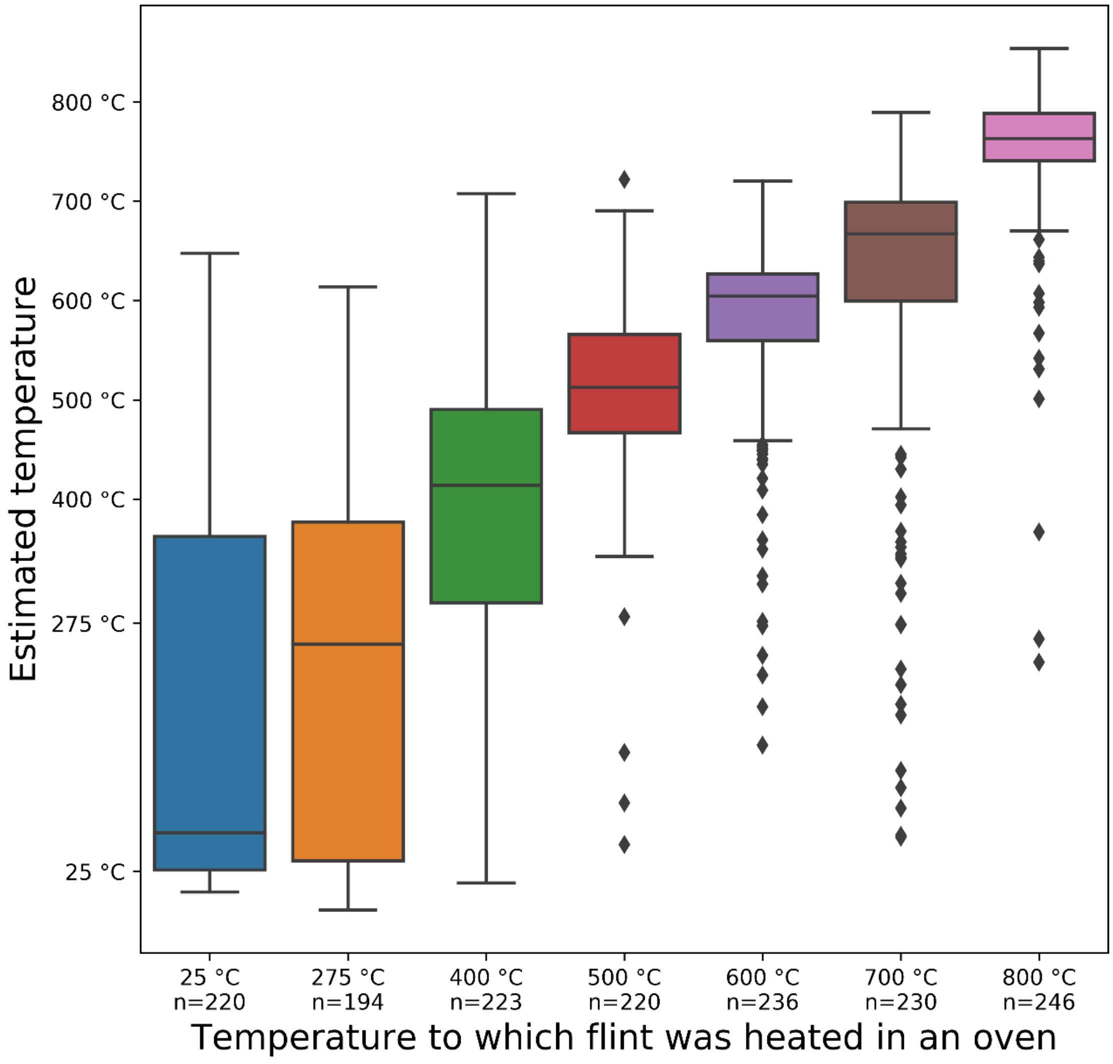

Fig. S6. Box plots of the temperature estimations for the validation experiment determined by the 1D-CNN model for each temperature category. The lines in each box represent the different quartiles, while the one inside the box is the median and the diamonds are outliers. Ten flint fragments were used for each temperature category (each from a different geological source). n represents the number of individual measurements (numerous measurements were taken from each flint fragment).

Interpretation of the validation experiment and confidence in temperature estimation

The 1D-CNN model succeeded in estimating the expected temperatures for the different temperature categories. Similar to our previous work using FC-ANN (8), we acknowledge that the 1D-CNN model performs better at higher temperatures. At lower temperature categories (room temperature and 275 °C), the calculated medians (64 °C, 254 °C) are close to the true values. However, the average value for the room temperature was calculated to be 189 °C. We assigned the category "room temperature" to flint samples that we did not heat in an oven.

We performed the Kruskal-Wallis statistical test (10) for all estimated temperature categories as well as between all pairs of estimated temperature categories. The distributions of estimated temperatures are statistically different for the different temperature categories. As expected, we found the largest *p*-value (0.016) between room temperature and 275 °C categories. Furthermore, a *p*-value of $1.5x10^{-14}$ was determined between estimated temperatures of categories of 275 °C and 400 °C. This *p*-value indicates high confidence in the separation between these temperatures categories. For compliance, we supply the *p*-values for all adjacent pairs of temperature categories: room temperature - 275 °C (0.016), 275-400 °C ($1.5x10^{-14}$), 400-500 °C ($6.0x10^{-20}$), 500-600 °C ($7.6x10^{-19}$), 600-700 °C ($2.6x10^{-16}$), 700-800 °C ($2.3x10^{-54}$).

Overall, temperature estimation of flint artifacts using our DL-based approach is limited for the lower temperatures. This has been previously attributed to the relatively small training dataset, structural/physical limitations, and/or flint's thermal history (8). We cannot discriminate their singular and combined influence on the estimated temperatures at the moment. Yet, statistical tests between pairs of temperature categories, in-depth understanding of flint's ultrastructure and components' spatial distribution, and simultaneous multispectral identification of non-linear

patterns support the idea that DL-based models can be reliably used to estimate the temperatures to which geological flint and flint–based artifacts have been exposed.

All code was written with Python and is available at the online public repository: https://github.com/fnatalio/Evron_Quarry. Specifically, Scikit-Learn (11) and Keras packages ((12) and https://github.com/fchollet/keras, 2015) were applied for machine learning and deep learning implementations, and Scipy (13) was applied for statistical and data analysis.

Table S2. List of flint-based artifacts measured by UV Raman spectroscopy and correspondent temperature estimation using two different machine-learning models [1D-CNN and FC-ANN(8)].

				1D-CNN		FC-ANN (8)	
**#**	**Flint artifact**	**Sub-square coordinates**	**Number of used measurements / number of total measurements**	**Average estimated temperature (°C)**	**Standard deviation, (°C)**	**Average estimated temperature, (°C)**	**Standard deviation, (°C)**
1	L5_6	yes	23 / 27	567.4	121.2	587.7	66.3
2	L5_239	no	22 / 24	549.5	45.6	568.9	40.0
3	L5_3	yes	21 / 27	418.4	213.0	486.5	143.6
4	L5_248	no	27 / 27	404.9	139.8	381.9	188.0
5	L5_244	no	27 / 27	303.5	103.9	307.7	118.8
6	L5_35	yes	27 / 27	287.9	165.8	438.6	162.9
7	L5_43	yes	22 / 27	185.0	102.2	206.1	135.0
8	L5_241	no	25 / 27	129.7	136.9	261.4	124.4
9	L5_26	yes	27 / 27	126.7	118.3	357.0	157.7
10	L5_235	no	27 / 27	87.8	122.7	158.8	164.4
11	K5_waste	no	27 / 27	561.8	57.9	495.7	87.4

12	K5_84	yes	26 / 27	380.9	169.7	474.7	115.9
13	K5_wis1	no	25 / 27	321.3	206.7	311.2	187.4
14	K5_121	no	22 / 27	243.6	128.9	357.9	160.2
15	K5_106	no	25 / 27	155.0	144.5	243.6	137.9
16	K5_99	yes	22 / 27	100.2	92.4	157.3	129.7
17	K5_wis2	no	24 / 27	97.5	147.7	186.7	124.0
18	K5_79	yes	27 / 27	92.2	85.8	188.0	117.6
19	K5_66	yes	25 / 27	91.6	111.6	145.8	129.7
20	K5_102	no	22 / 27	38.3	52.0	102.6	101.8
21	K5_14	yes	27 / 27	31.6	34.2	129.4	119.0
22	K3_18	yes	26 / 27	651.8	106.2	631.2	131.9
23	K3_40	yes	27 / 27	342.2	96.7	174.3	135.7
24	K3_28	yes	27 / 27	333.0	189.0	400.9	164.8
25	K3_14	yes	25 / 27	224.3	150.8	324.0	128.7
26	K3_9	yes	27 / 27	192.5	103.9	338.7	109.0

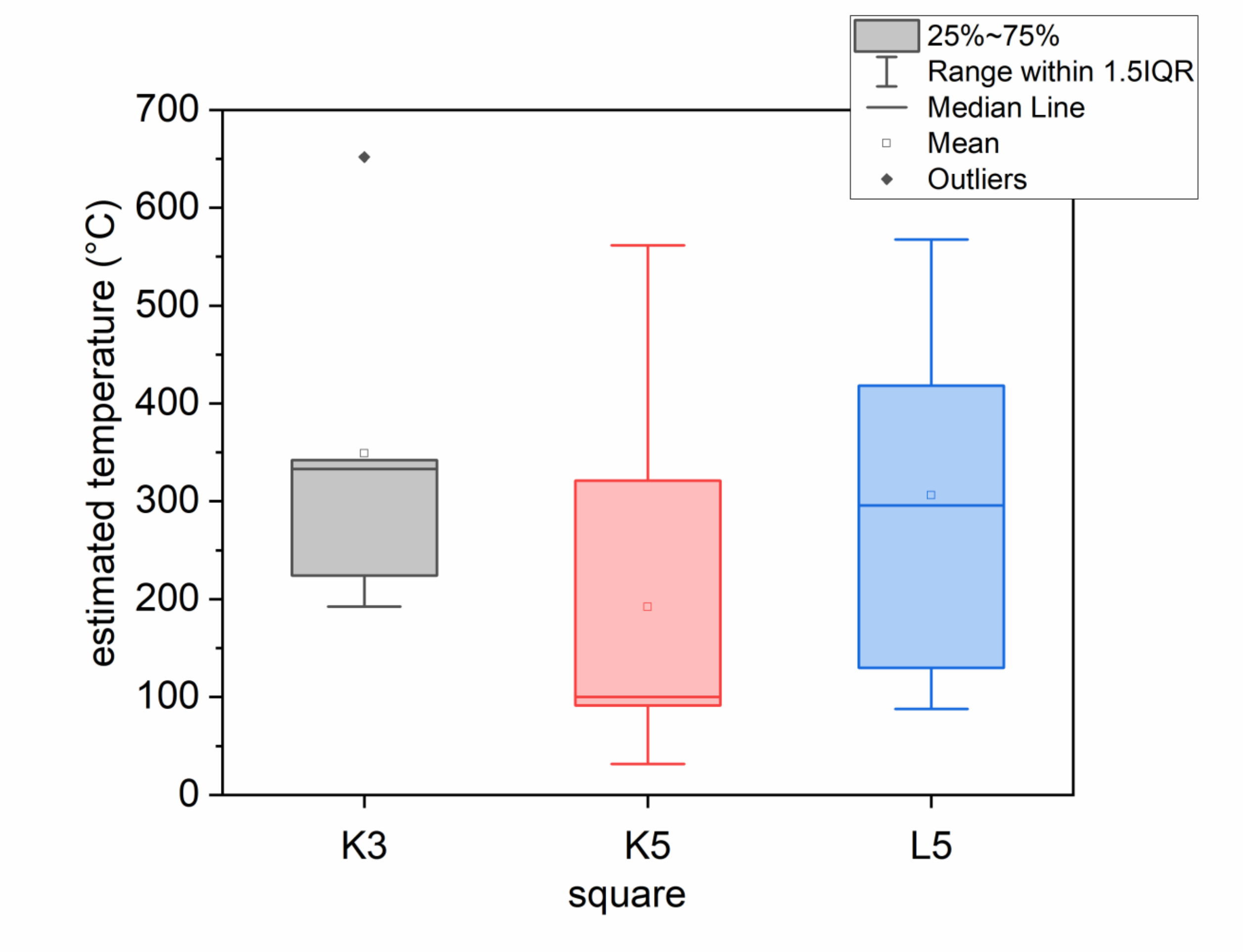

Fig. S7. Boxplot of estimated temperatures using deep learning model [1D-CNN] (Table S2) for lithic artifacts from K3, K5, and L5 squares. The number of samples per square: K3 – 5, K5 – 11, L5 – 10.

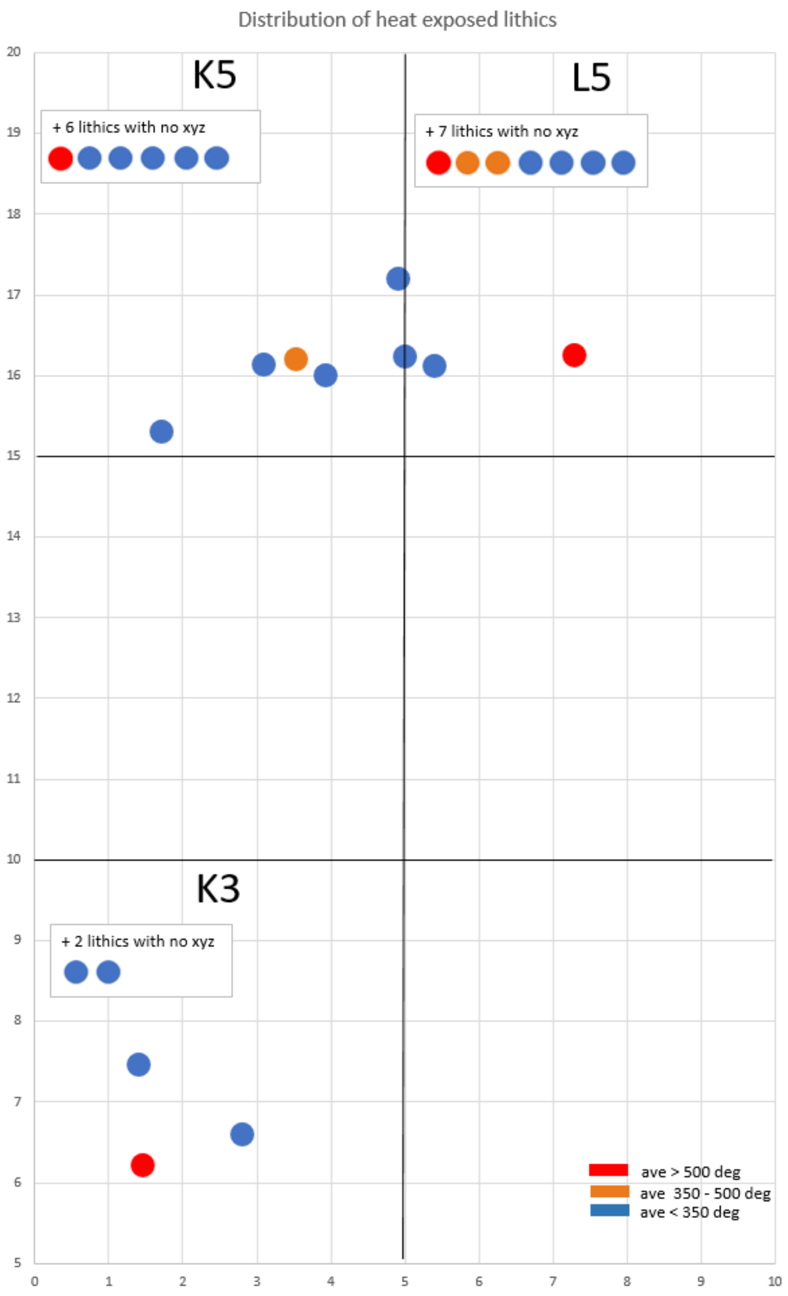

Fig. S8. Spatial distribution in 2D of the lithics color-coded according to the estimated temperatures showing a heterogeneous distribution (each square [e.g. K5, L5] is 5x5 m).

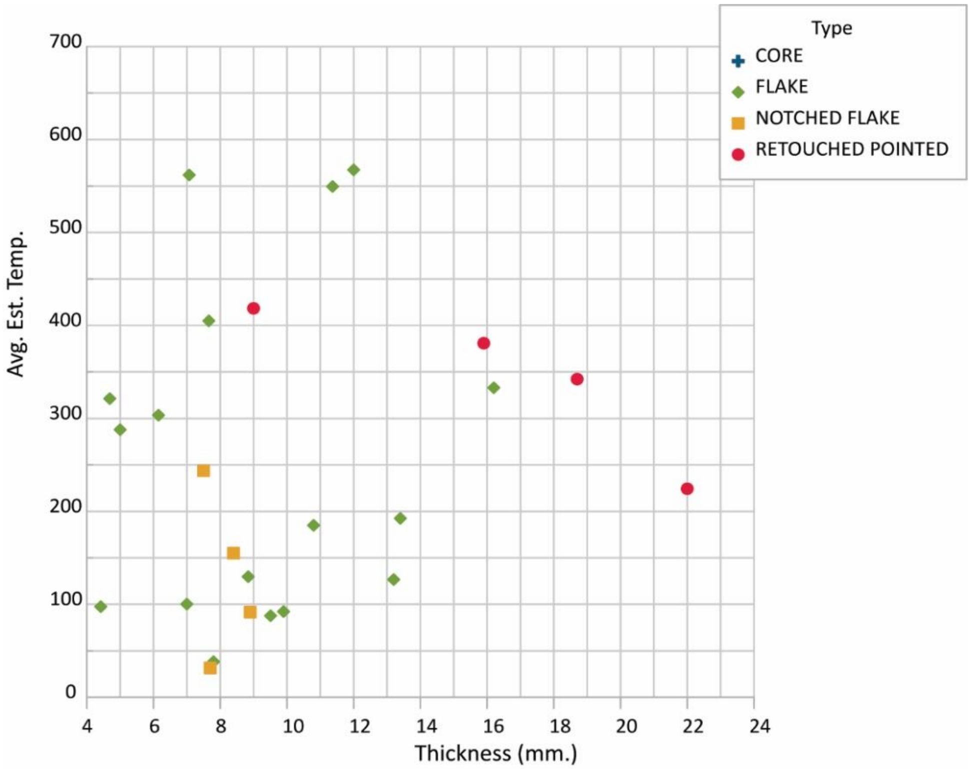

Type
CORE
FLAKE
NOTCHED FLAKE
RETOUCHED POINTED
Avg. Est. Temp.
0
100
200
300
400
500
600
700
4
6
8
10
12
14
16
18
20
22
24
Thickness (mm.)

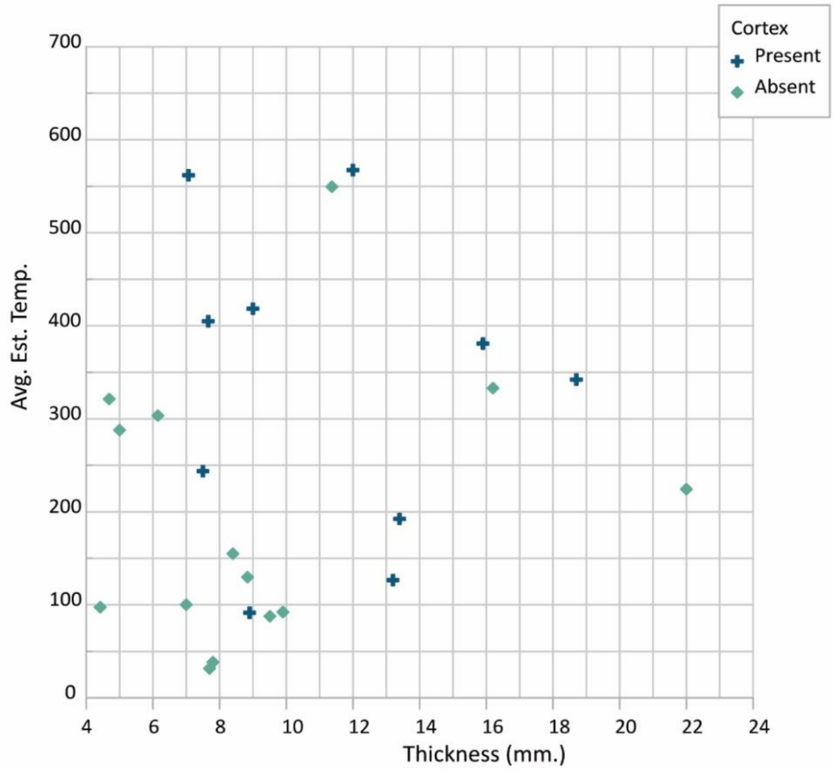

Cortex
Present
Absent
Avg. Est. Temp.
0
100
200
300
400
500
600
700
4
6
8
10
12
14
16
18
20
22
24
Thickness (mm.)

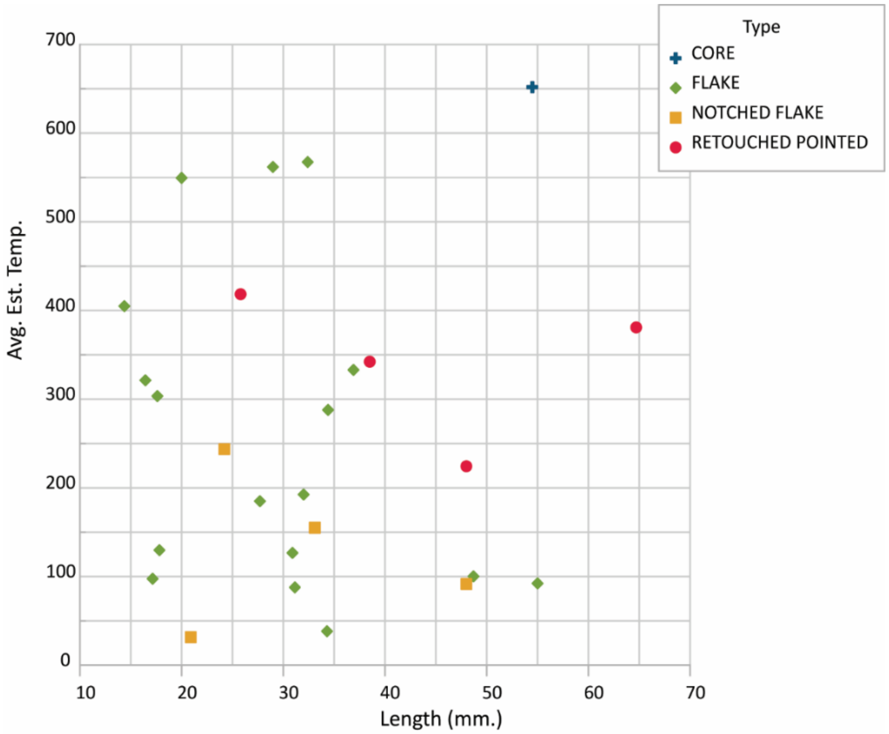
Type
CORE
FLAKE
NOTCHED FLAKE
RETOUCHED POINTED
Avg. Est. Temp.
0
100
200
300
400
500
600
700
10
20
30
40
50
60
70
Length (mm.)

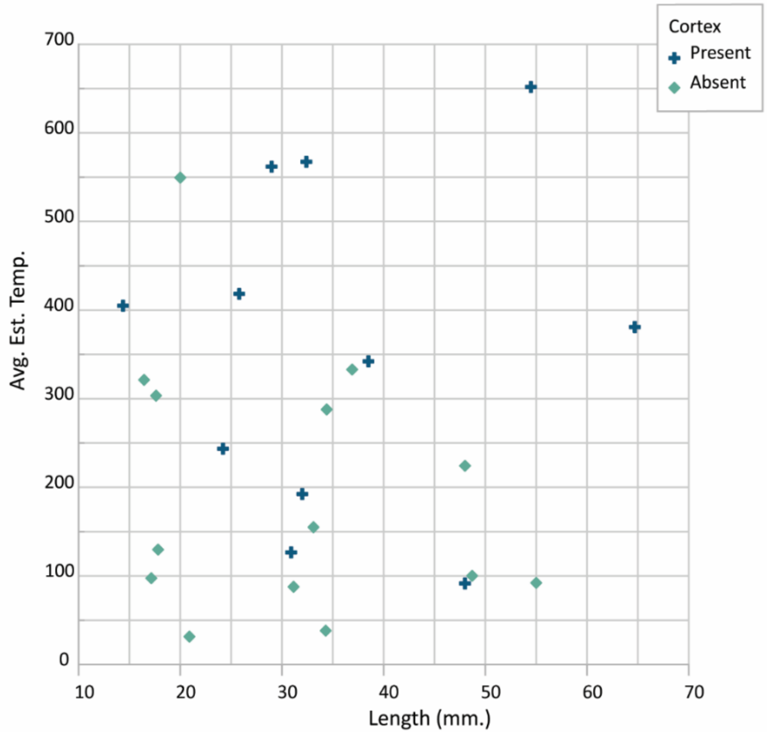
Cortex
Present
Absent
Avg. Est. Temp.
0
100
200
300
400
500
600
700
10
20
30
40
50
60
70
Length (mm.)

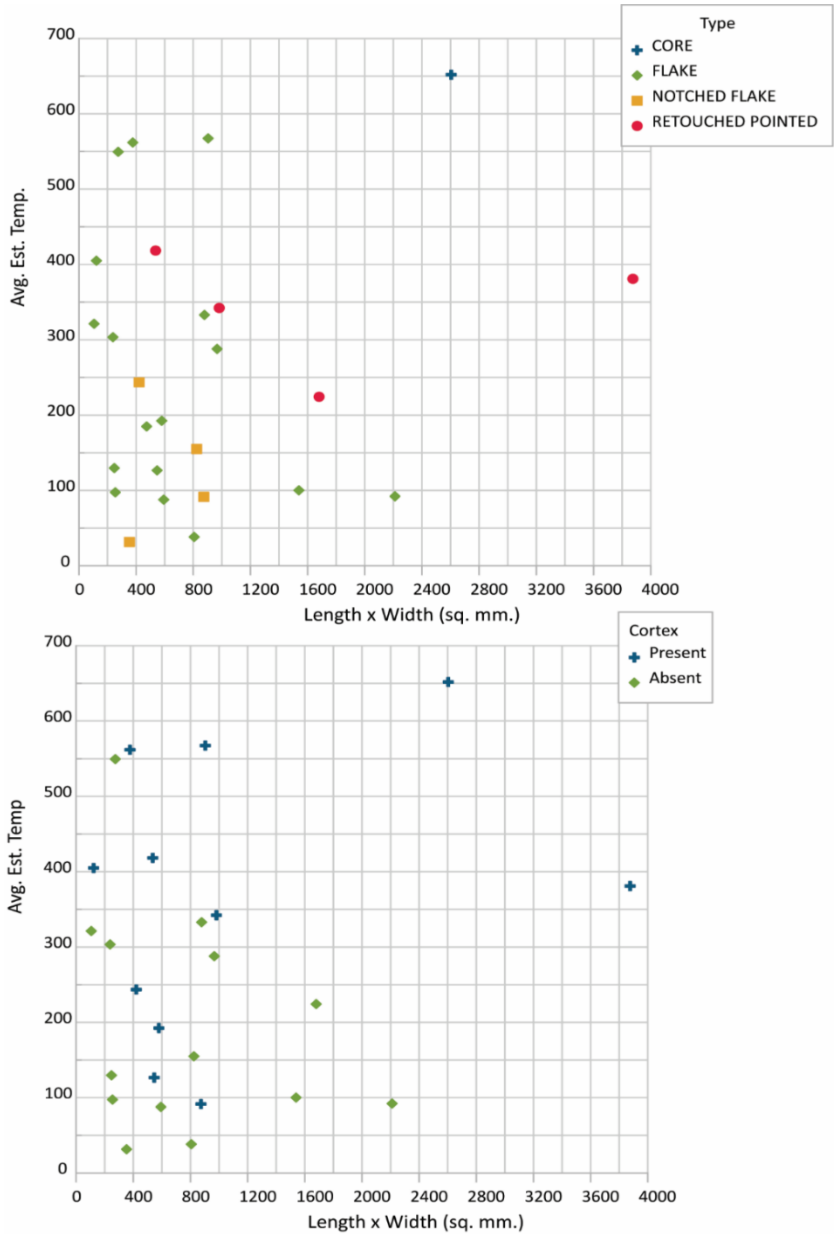

Fig. S9. Plots of lithics estimated temperatures versus different physical parameters (i.e., thickness, length *x* width, length. No correlation is found between the type of artifact, size of artifact (length, thickness, and surface area calculated as length x width), and temperature of heating, nor is there a correlation between the temperature of heating and presence or absence of cortex. Note that the absence of retouched pointed artifacts at either high or low temperature is likely due to sampling size.

The Evron Quarry faunal assemblage

Fauna from Evron Quarry was first identified by Georg Haas (14, 15) and listed as comprising molars of proboscideans, ruminants, hippopotamus, and a large endemic phacochoerid. Additional finds from the first excavations in the 1970s were identified by Eitan Tchernov (16), while the most comprehensive taxonomic description of all remains recovered from the excavation as well as sundry items collected from the quarry floor, appears in Tchernov *et al.* (1994)(17). This faunal list documents 12 species and includes medium to large-sized herbivores; a large bovid, identified as probable aurochs (*Bos* cf. *primigenius*), a species of gazelle (*Gazella* sp.), cervids (*Cervus* cf. *elpahus* and *Capreolus* sp.) and possibly also hartebeest (cf. *Alcelaphus*); an endemic Suid (*Kolpocherous evronensis*), as well as mega-herbivores including two species of proboscidean – *Stegodon*, *Elephas* – and hippopotamus (*H. amphibius*). In addition, isolated remains of rodents, freshwater turtle, and possibly a hyaena were identified (17). Most faunal items have only square numbers, and information on sub-squares is lacking such that they could not be plotted within the 5 x 5 m grid. Some carnivore-derived damage was evident on the bones, but no anthropogenic-derived damage (cut marks or percussion fractures) has been reported. A comprehensive taphonomic study of the assemblage is underway, including examining the unidentified bone fragments. Tchernov *et al*. (1974)(17) noted that the fauna represents a biogeographical mixture resulting from biotic exchanges between the Ethiopian, Oriental, and Palaearctic regions. One question which remains unanswered is whether the Evron faunal assemblage represents a new dispersal event from Asia or Africa or the continuation of an earlier dispersal event related to the ‘Ubeidiya faunal assemblage dated to *ca.* 1.4 Mya.

A random sample of 87 faunal remains out of over 200 recovered items was analyzed for this study (Table S3, Fig. S10), which derived solely from known squares in the 1976-77 excavations. They include at least seven samples (# 1-7 in Table S3 and Fig. S10) from what appears to have been a single proboscidean tusk (named Tusk 300) found in square L5 that broke up into several pieces (Table S4, Fig. S11). FTIR results show that this tusk was burnt due to the presence of the peak at 630 cm^{-1} (Fig. S12). Given their small size, faunal samples displaying 630 cm^{-1} peak were examined under SEM to verify our initial visual identification and confirm if they represented bone or tusk (Fig. S14). All burnt fragments are tusk, and we do not discard the possibility of belonging to the same tusk.

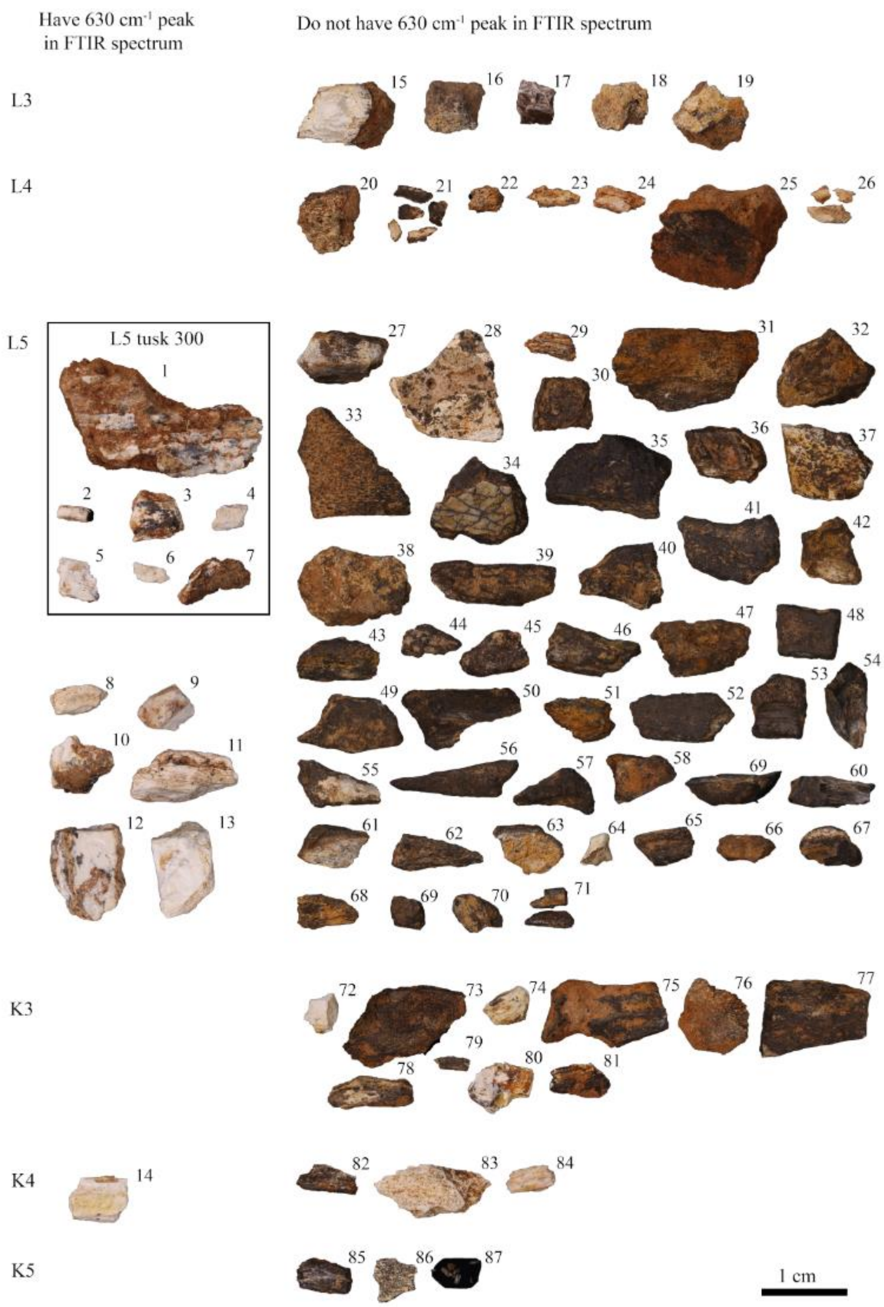

Fig. S10. Photograph of all faunal remains analyzed in this study from Unit 4 Evron Quarry, 1976-77 excavations. Fragments of tusk listed as L5_300 used for the FTIR analysis are displayed in the square on the top left. See Fig. S11 for images of the whole preserved pieces of the tusk. Note that some fragments that were found not to have been exposed to high

temperatures (e.g., #15, #72, #80, #83) are white; thus white color does not represent exposure to high temperatures in this assemblage.

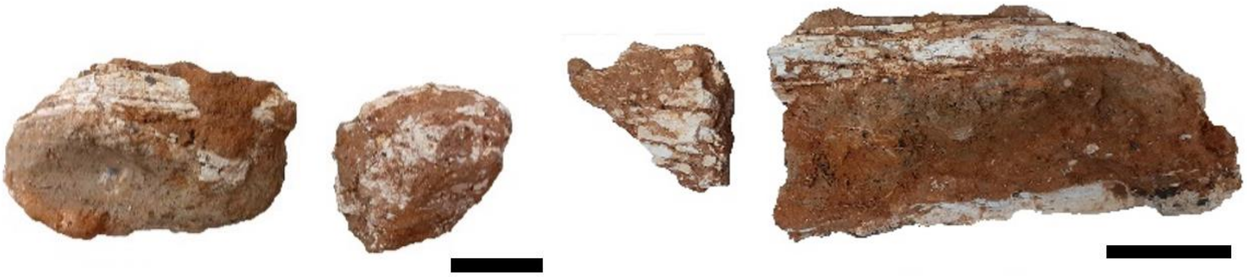

L5_300_(1)

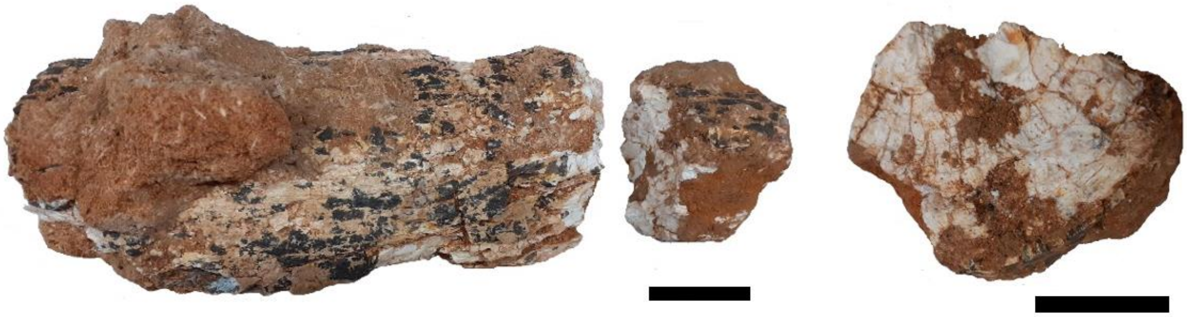

L5_300_(2) (left: side view; right: cross sectional view)

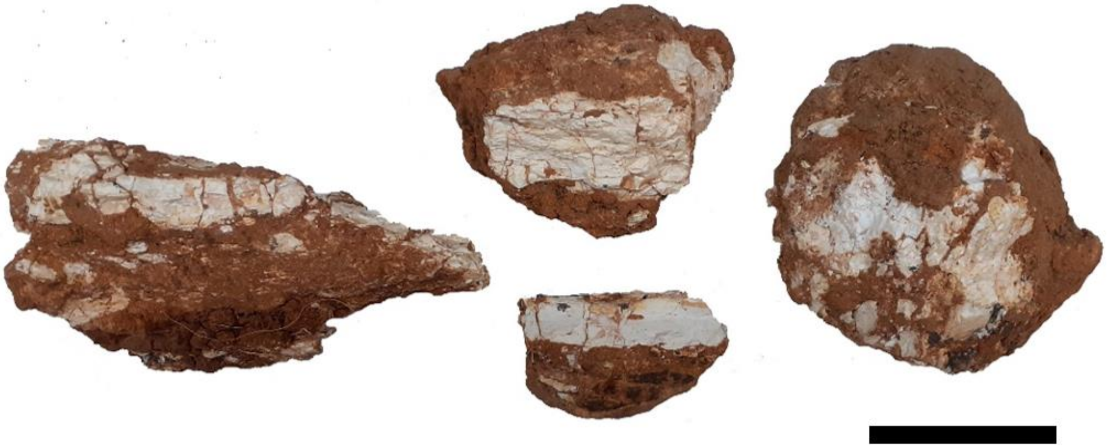

L5_300_(3)

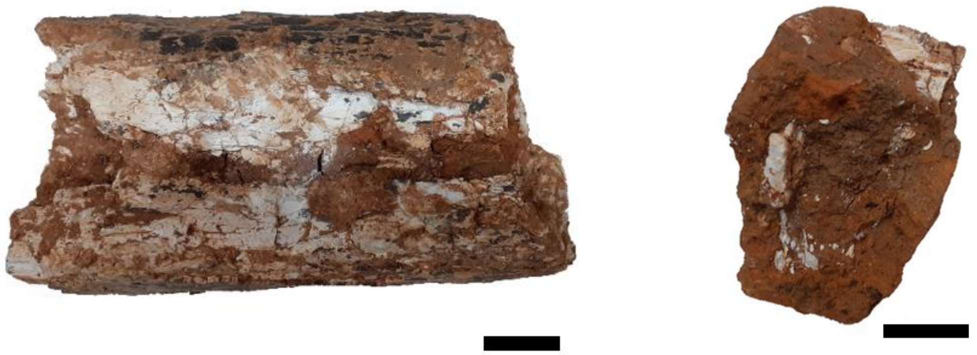

L5_300_box7 L5_300 (7)

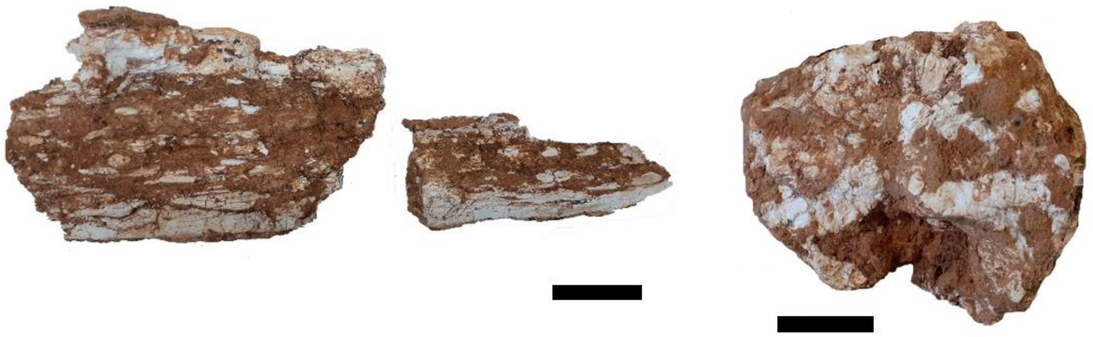

L5_300_(8) L5_300_box3

Fig. S11. Photographs of the square L5_300 tusk fragments. Scale bars: 3 cm. Note the adhering red *Hamra* sediment.

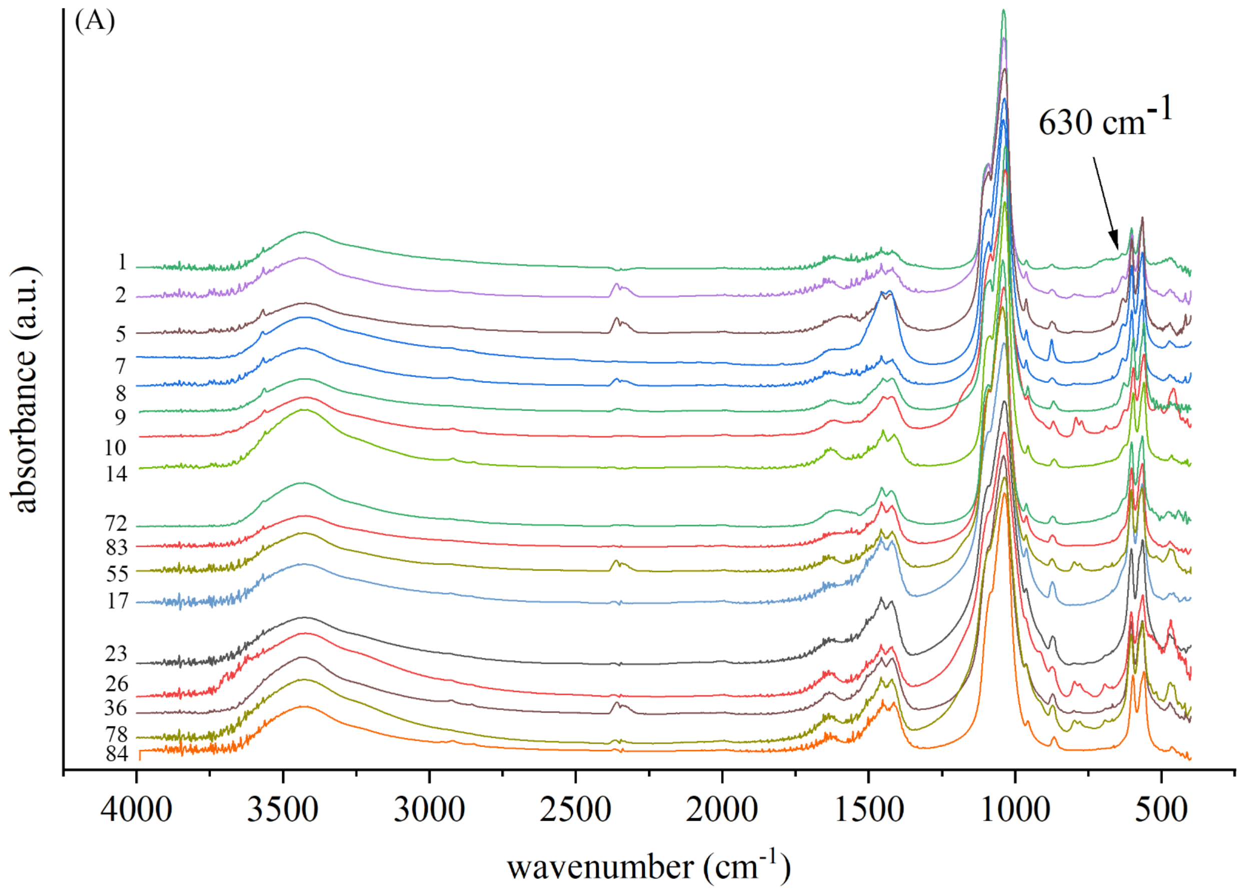

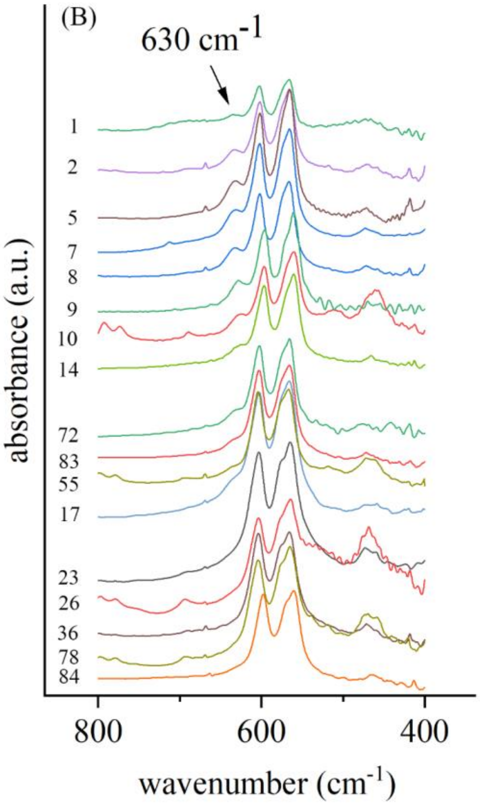

Fig. S12. Representative Fourier-transform Infrared spectroscopy (FTIR) analysis of faunal fragments collected from different squares from Unit 4 (Evron Quarry) collected during the 1976-77 excavations. (A) corresponding full spectra. (B) close-up of the region containing the 630 cm^{-1} peak used for identifying heating to high temperatures. Sample numbers correspond to numbering in Table S3. The top-most eight spectra (#1, 2, 5, 7, 8, 9, 10, and 14) represent samples with a prominent 630 cm^{-1} hydroxylation peak which we, therefore,

interpreted as fauna exposed to temperatures above 600 °C. Note that sample #14 has a relatively small peak – this is the smallest peak among the samples measured. Samples #72, 83, 55, and 17 have a shoulder at 630 cm^{-1}. The lowermost samples (#23, 26, 36, 78, and 84) are examples of faunal fragments with no peak at 630 cm^{-1}; these samples have not been exposed to high temperatures.

Comments on tusk and FTIR

Bone, teeth, and tusk differ in their architecture, characterized by a high degree of structural hierarchy from the macro-, meso-, micro- to the nanoscale (18). Bone is composed of different types (spongy/trabecular and compact bone) that differ in their structure. Teeth are composed of enamel and dentin, that structurally differ from each other and from bone. Tusk is composed mainly of dentin with a similar structure to teeth dentin (i.e., dentinal tubules) but with an additional and unique structural feature called Schreger lines (19). From the chemical point of view, however, the composition of dentin in teeth and tusk is similar (20). All bone, teeth and tusk are composed of non-stoichiometric carbonated OH-deficient hydroxylapatite chemically represented by $Ca_5(PO_4)_{3-x}(CO_3)_xOH$ (21-24) with small variations attributed to biological variability even within the same species. Thus, one can hypothesize that dentin would undergo the same hydroxylation change as bone after exposure to heat. Unfortunately, to the best of our knowledge, there is no available literature focusing on the 630 cm^{-1} peak evolution in ancient or modern ivory as a function of heating. However, Lim et al. (25) used FTIR to characterize extracted human tooth powder sintered at temperatures in the range of from 600 °C to 1200 °C where they found the presence of the 630 cm^{-1} peak in all samples, which they attributed to the presence of heat-induced addition of hydroxyl groups as reported for bone (23, 26) supporting our hypothesis that dentin also undergoes hydroxylation in the same temperature range.

The advantage of focusing on the 630 cm^{-1} band rather than other peaks is that it is exclusively attributed to a libration vibration band from OH groups added during heating and not influenced by diagenesis as in the case of bands diagnostic of crystallinity. The use of 630 cm^{-1} band in bones is well documented and confirmed by an extensive body of literature based on the pioneering work of Figueiredo *et al.*(26) and Rey *et al.*(23). Because these are very old samples, we performed X-ray diffraction analysis on several bones and tusk from Evron Quarry and confirmed that they have the same chemical composition and no diagenetic phases were found (Fig. S13).

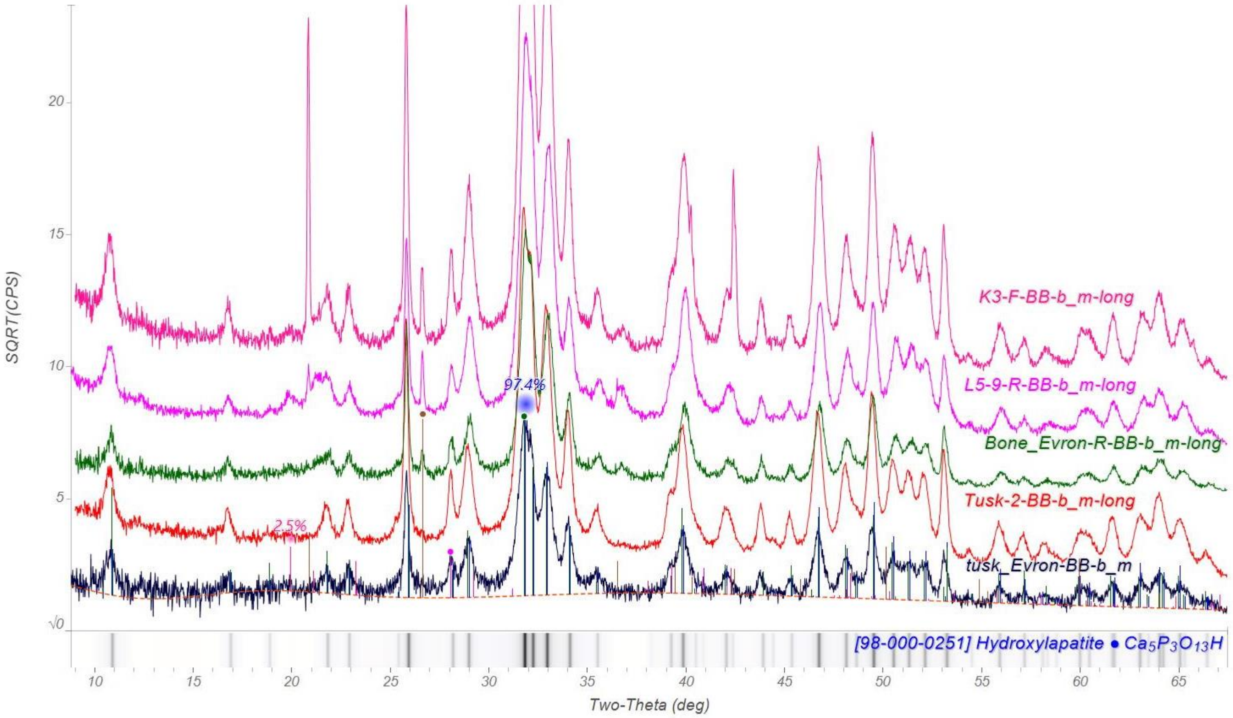

Fig. S13. Powder XRD comparative analysis between several bones and tusk unearthed from Evron Quarry archaeological site showing that they are chemically similar.

Experimental note:

Diffraction measurements were carried out in reflection geometry using an Ultima III (Rigaku, Japan) diffractometer equipped with a sealed Cu anode X-ray tube operating at 40 kV and 40 mA. A bent graphite monochromator and a scintillation detector were aligned in the diffracted beam. θ/2θ scans were performed under specular conditions in the Bragg–Brentano mode with variable slits. The 2θ scanning range was 10–70° with step size and scan speed of 0.02° and 0.5°/min, respectively. Phase analysis was performed using the PDF-4+ 2020 database (ICDD) and Jade Pro software (Materials Data, Inc.).

Several studies indicated that magnesium (Mg^{2+}) in dentin was a key trigger ion material that accelerates the thermal decomposition of dentin (27). We conducted Elemental Dispersive X-rays analysis of the bone and tusk unearthed from Evron Quarry. We found the same Mg atomic concentration percentage average of 0.1% (based on triplicate measurements) for bone and tusk.

Table S3. List of faunal remains and FTIR results summary.

#	Sample	Color	Peak at 630 cm^{-1}	Exposed to high temperature
1	L5_300_(1)	white	yes	yes
2	L5_300_(2)	white	yes	yes
3	L5_300_(3)	white	yes	yes
4	L5_300_(7)	white	yes	yes
5	L5_300_(8)	white	yes	yes
6	L5_300_box7	white	yes	yes
7	L5_300_box3	white	yes	yes
8	L5_(1)	white	yes	yes
9	L_5_small_bag	white	yes	yes
10	L5	white	yes	yes
11	L5_wis1	white	yes	yes
12	L5_wis4	white	yes	yes
13	L5_wis7	white	yes	yes
14	K4_H_(3)	white	small peak	yes
15	L3_Y_(I)_HT_14-93-14-85	white	no	no
16	L3_Y_14-93-14-85_(III)	white + dark*	no	no
17	L3_H	white + dark	shoulder	no
18	L3_Y_HT14-93-14-85_12-7-77	yellow	no	no

19	L3_Y_HT14-93_63	white	no	no
20	L4_W_(I)_15.25	yellow	no	no
21	L4_V_(I)	white + dark	no	no
22	L4_V_(4)	dark	no	no
23	L4_V_(5)	white + dark	no	no
24	L4_P_(2)	dark	no	no
25	L4_W_(2)	white	no	no
26	L4_Q_(1)	white	no	no
27	L5_wis2	white	no	no
28	L5_wis3	white	no	no
29	L5_wis5	grey	no	no
30	L5_wis6	dark	no	no
31	L5_wis8	dark	no	no
32	L5_wis9	dark	no	no
33	L5_wis10	dark	no	no
34	L5_wis11	dark	no	no
35	L5_wis13	dark	no	no
36	L5_wis14	dark	no	no
37	L5_wis15	dark	no	no
38	L5_wis16	dark	no	no
39	L5_wis17	dark	no	no
40	L5_wis18	dark	no	no

41	L5_wis19	dark	no	no
42	L5_wis20	dark	no	no
43	L5_wis21	dark	no	no
44	L5_wis22	dark	no	no
45	L5_wis23	dark	no	no
46	L5_wis24	dark	no	no
47	L5_wis25	dark	no	no
48	L5_wis26	dark	no	no
49	L5_wis27	dark	no	no
50	L5_wis28	dark	no	no
51	L5_wis29	dark	no	no
52	L5_wis30	dark	no	no
53	L5_wis31	dark	no	no
54	L5_wis32	dark	no	no
55	L5_wis33	dark	shoulder	no
56	L5_wis34	dark	no	no
57	L5_wis35	dark	no	no
58	L5_wis36	dark	no	no
59	L5_wis37	dark	no	no
60	L5_wis38	dark	no	no
61	L5_wis39	dark	no	no
62	L5_wis40	dark	no	no

63	L5_wis41	dark	no	no
64	L5_wis42	dark	no	no
65	L5_wis44	dark	no	no
66	L5_wis45	dark	no	no
67	L5_wis46	white + dark	no	no
68	L5_wis47	dark	no	no
69	L5_wis49	dark	no	no
70	L5_wis50	dark	no	no
71	L5_23	dark	no	no
72	K3_60	white	shoulder	no
73	K3_71	dark	no	no
74	K3_59	white	no	no
75	K3_E_7-4-74	dark	no	no
76	K3	dark	no	no
77	K3_bag	dark	no	no
78	K3_F_HT14-50	dark	no	no
79	K3_E	white	no	no
80	K3_E_(9)	white	no	no
81	K3_59	white + dark	no	no
82	K4_T	dark	no	no
83	K4_D_(I)	white	shoulder	no
84	K4_C_(I)	white	no	no

85	K5	dark	no	no
86	K5_89	white + dark	no	no
87	K5_96	dark	no	no

* “dark” here and in other instances indicate different hues of brown, grey, and black

Table S4. Distribution of faunal samples analyzed per excavation square.

**Square**	**N faunal samples analyzed**	**N faunal samples exposed to high temperature**
**L3**	5	0
**L4**	7	0
**L5**	57*	13*
**K3**	10	0
**K4**	4	1
**K5**	3	0
**Total**	92*	15*

*at least seven belong to a single tusk, “Tusk 300”.

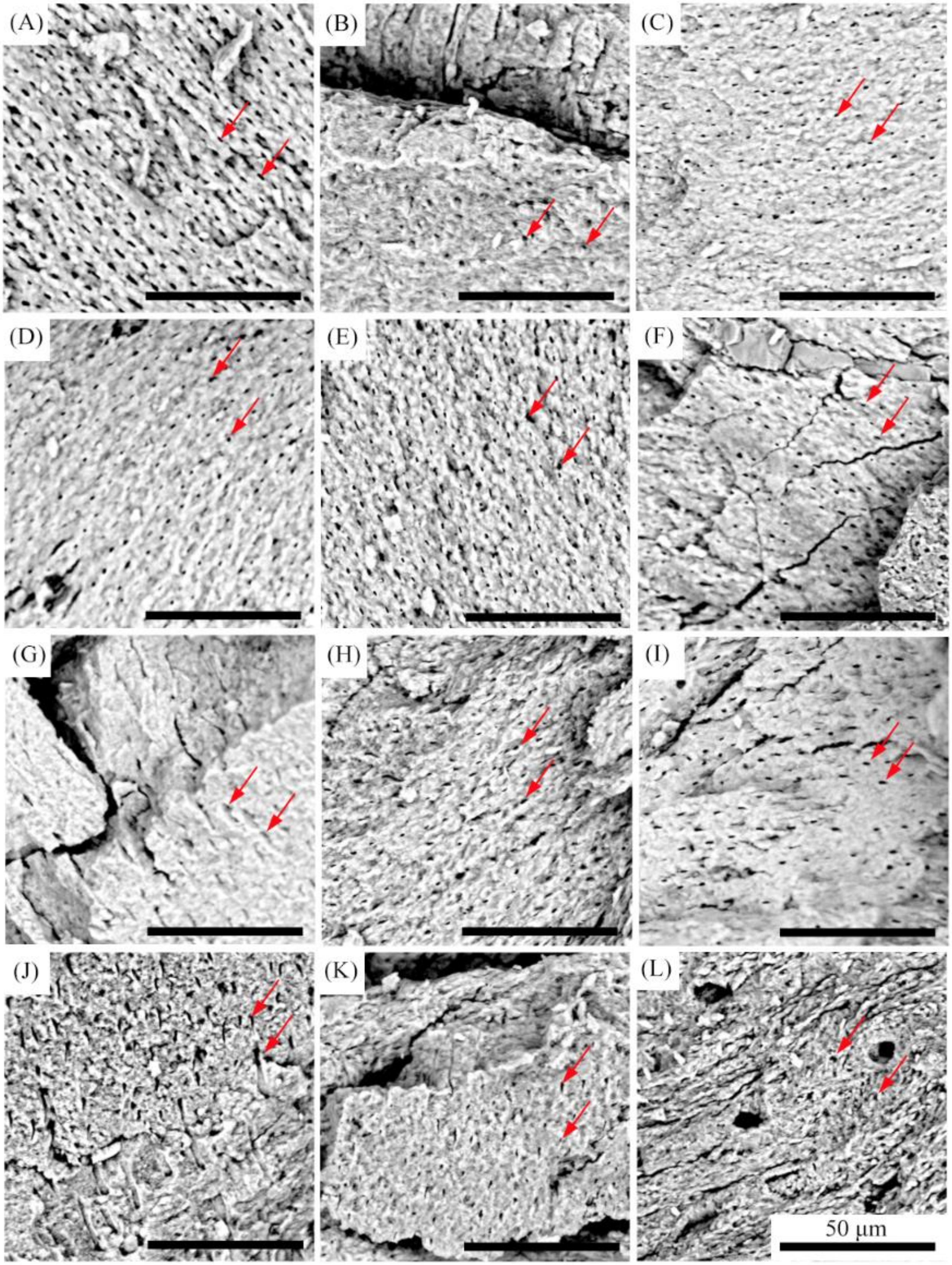
(A)
(B)
(C)
(D)
(E)
(F)
(G)
(H)
(I)
(J)
(K)
(L)
50 μm

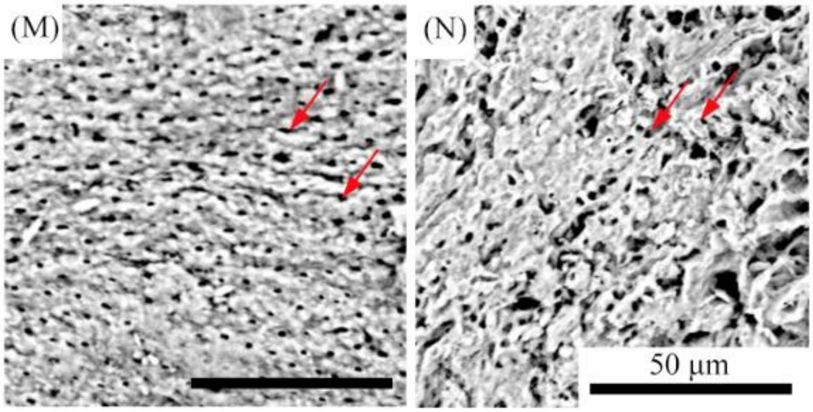

Fig. S14. Representative scanning electron images of tusk displaying an explicit 630 cm^{-1} peak in the FTIR spectrum attributed to hydroxylation of the mineral structure and considered burnt. (A) L5_300_(1), (B) L5_300_(2), (C) L5_300_(3), (D) L5_300_(7), (E) L5_300_(8), (F) L5_300_box7, (G) L5_300_box3, (H) L5_(1), (I) L_5_small_bag, (J) L5, (K) L5_wis1, (L) L5_wis4, (M) L5_wis7, (N) K4_H_(3). Note the dentinal tubules (marked with red arrows) typical of the tusk (28). We do not exclude the possibility that all burnt tusk fragments derive from the same tusk.

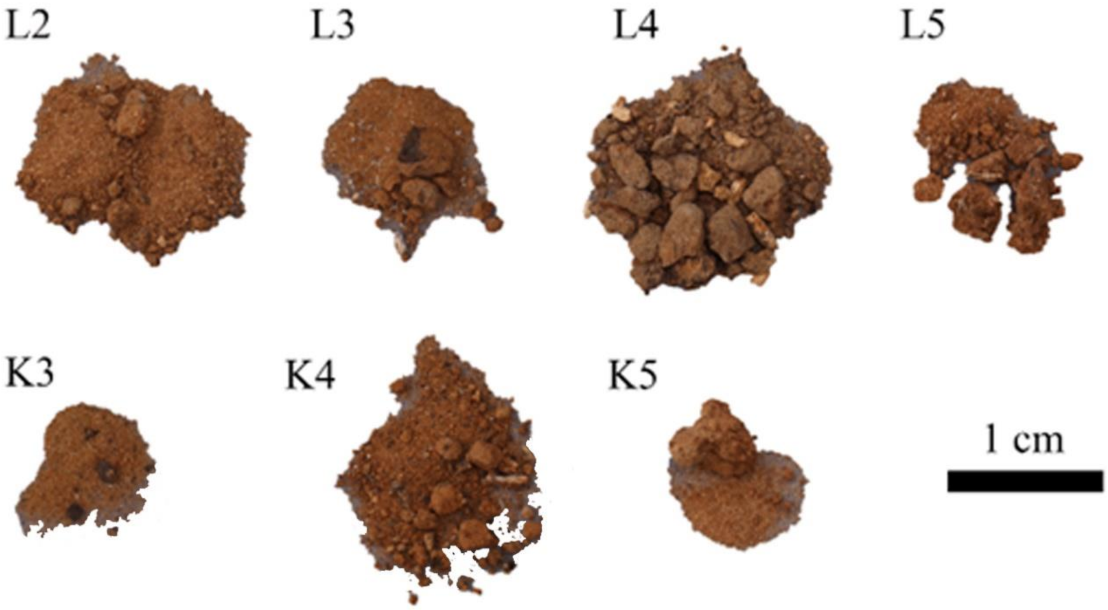

Fig. S15. Representative photographic images of the sediments collected from different squares (Evron Quarry), 1976-77 excavations Unit 4. All sediments display similar red coloration typical of *Hamra*.

Table S5. Fourier-Transform Infrared spectroscopy analysis (FTIR) analysis of sediments collected from different squares from Units 4 (Evron Quarry) 1976-77 excavations and associated with faunal remains (see adhering sediment to the burnt tusk in Fig. S11).

#	Sample ID	Peaks at (-OH region) 3570 cm^{-1} (structural water)	Main clay peaks wavenumber	burnt
1	L5_300_(1)_sed	yes	1036	no
2	L5_300_(2)_sed	yes	1035	no
3	L5_300_(3)_sed	yes	1035	no
4	L5_300_(7)_sed	yes	1035	no
5	L5_300_(8)_sed	yes	1036	no
6	L5_300_box7_sed	yes	1035	no
7	L5_300_box3_sed	yes	1035	no
8	L5_(1)_sed	yes	1035	no
9	L_5_small_bag_sed	yes	1035	no
10	L5_sed	yes	1036	no
11	L3_Y_(I)_HT14-93-14-85_sed	yes	1035	no
12	L3_Y_14-93-14-85_(III)_sed	yes	1036	no
13	L3_H_sed	yes	1032	no
14	L3_Y_HT14-93-14-85_12-7-77_sed	yes	1032	no
15	L3_Y_HT14-93_63_sed	yes	1035	no
16	L4_W_(I)_15-25_sed	yes	1035	no
17	L4_V_(I)_sed	yes	1035	no
18	L4_V_(4)_sed	yes	1035	no

19	L4_V_(5)_sed	yes	1036	no
20	L4_P_(2)_sed	yes	1035	no
21	L4_W_(2)_sed	yes	1032	no
22	L4_Q_(1)_sed	yes	1032	no
23	K3_71_sed	yes	1035	no
24	K3_59_sed	yes	1035	no
25	K3_E_7-4-74_sed	yes	1035	no
26	K3_sed	yes	1035	no
27	K3_E_sed	yes	1035	no
28	K3-E 13/94-13.86 sed	yes	1036	no
29	K3_E_(9)_sed	yes	1036	no
31	K4_T_sed	yes	1035	no
32	K4_D_(I)_sed	yes	1035	no
33	K4_C_(I)_sed	yes	1036	no
34	K5_96_sed	yes	1035	no

Sediments

We used FTIR to assess whether the sediments (n= 34) associated with fauna from different excavation squares had been exposed to heat above 400 °C. The analyzed sediments were either lumps of sediment that had been packed in with the fauna on-site or were directly adhering to faunal remains. All sediments display similar red coloration typical of *Hamra* (Fig. S15) and consist predominantly of α-quartz and clay. In some cases, minor quantities of calcite can be found. No other phases such as dahllite, amorphous silica/opal were identified. We found that all isolated clay fractions show the presence of clay structural water (around the 3600 cm^{-1} region) and unshifted clay main peak (1032-1036 cm^{-1}) (Fig. S16, Table S5), suggesting that the sediments had not been exposed to temperatures above 400°C (29) nor had they been exposed to heat for prolonged periods (30). Except for two sediment samples (K5_96_sed and L5_300_box7_sed), the clay was separated from α-quartz by density using sodium polytungstate solution (see Material and Method section above). This protocol was applied due to α -quartz shadowing the clay peak.

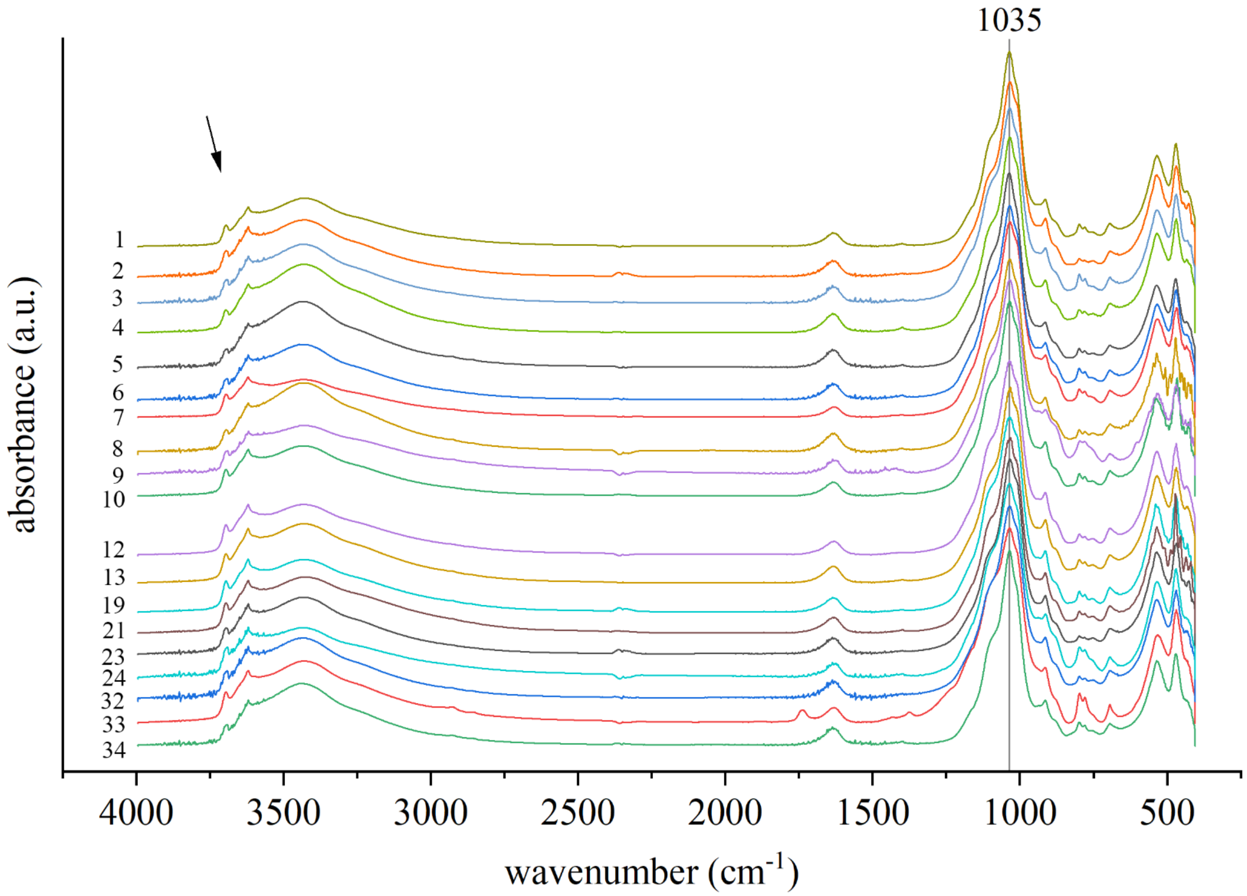

Fig. S16. Representative Fourier-transform infrared spectroscopy analysis (FTIR) of the clay fraction of sediments collected from different squares from Units 4-5 (Evron Quarry) – 1976-77 excavation. All sediment samples have the clay main peak located at wavenumbers 1032-1036 cm^{-1} and display clay structural water peaks (around the 3600 cm^{-1} region highlighted by a black arrow), suggesting that the sediments were not exposed to temperatures higher than 400 °C (29) or for prolonged periods (30). Sample numbers correspond to sample ID numbers in Table S5. Samples #1-10 represent sediments associated with burnt tusk fragments (Fig. S11). All other IR spectra displayed here represent two sediment samples from each excavation square.

References

1. A. Ronen, The lower palaeolithic site Evron-Quarry in western Galilee, Israel. *Sonderveröffentlichungen Geologisches Institut der Universität zu Köln* **82**, 187-212 (1991).
2. A. Ronen, J. M. Burdukiewicz, The small tools of Evron-Quarry, western Galilee, Israel. *BAR International Series* **1115**, 113-120 (2003).
3. M. Chazan, Butchering with small tools: the implications of the Evron Quarry assemblage for the behaviour of Homo erectus. *Antiquity* **87**, 350 (2013).
4. D. Gilead, M. Israel, An early palaeolithic site at Kefar Menahem preliminary report. *Tel Aviv* **2**, 1-12 (1975).
5. Y. Zaidner, R. Yeshurun, C. Mallol, Early Pleistocene hominins outside of Africa: recent excavations at Bizat Ruhama, Israel. *PaleoAnthropology* **2010**, 162-195 (2010).
6. Y. LeCun, Y. Bengio, Convolutional networks for images, speech, and time series. *The handbook of brain theory and neural networks* **3361**, 1995 (1995).
7. I. Goodfellow, Y. Bengio, A. Courville, *Deep learning* (MIT press, 2016).
8. A. Agam, I. Azuri, I. Pinkas, A. Gopher, F. Natalio, Estimating temperatures of heated Lower Palaeolithic flint artefacts. *Nature Human Behaviour* **5**, 221-228 (2021).
9. K. J. Kingma, R. J. Hemley, Raman spectroscopic study of microcrystalline silica. *American Mineralogist* **79**, 269-273 (1994).
10. W. H. Kruskal, W. A. Wallis, Use of Ranks in One-Criterion Variance Analysis. *Journal of the American Statistical Association* **47**, 583-621 (1952).
11. F. Pedregosa *et al.*, Scikit-learn: Machine learning in Python. *Journal of machine learning research* **12**, 2825-2830 (2011).
12. F. Chollet, *Deep Learning mit Python und Keras: Das Praxis-Handbuch vom Entwickler der Keras-Bibliothek* (MITP-Verlags GmbH & Co. KG, 2018).
13. P. Virtanen *et al.*, SciPy 1.0: fundamental algorithms for scientific computing in Python. *Nature methods* **17**, 261-272 (2020).
14. A. Issar, U. Kafri, The discovery of a Pleistocene mammalian fauna and artifacts at "Evron" Western Galilee. *Israel Journal of Earth-Sciences* **18** (1969).
15. G. Haas, Metridiochoerus evronensis n. sp. a new Middle Pleistocene Phacochoerid from Israel. *Israel Journal of Zoology* **19**, 179-181 (1970).
16. M. W. Prausnitz, A. Ronen, Early Acheulian Site in the Evron Quarry. *Israel Exploration Journal* **2**, 7 (1977).
17. E. Tchernov, L. K. Horwitz, A. Ronen, A. Lister, The faunal remains from Evron Quarry in relation to other Lower Paleolithic hominid sites in the southern Levant. *Quaternary Research* **42**, 328-339 (1994).
18. P. Fratzl, R. Weinkamer, Nature's hierarchical materials. *Progress in materials Science* **52**, 1263-1334 (2007).
19. M. Albéric, A. Gourrier, W. Wagermaier, P. Fratzl, I. Reiche, The three-dimensional arrangement of the mineralized collagen fibers in elephant ivory and its relation to mechanical and optical properties. *Acta Biomaterialia* **72**, 342-351 (2018).
20. I. Reiche, E. Chalmin, Synchrotron radiation and cultural heritage: combined XANES/XRF study at Mn K-edge of blue, grey or black coloured palaeontological and archaeological bone material. *Journal of Analytical Atomic Spectrometry* **23**, 799-806 (2008).

21. J. D. Pasteris *et al.*, Lack of OH in nanocrystalline apatite as a function of degree of atomic order: implications for bone and biomaterials. *Biomaterials* **25**, 229-238 (2004).
22. C. K. Loong *et al.*, Evidence of hydroxyl-ion deficiency in bone apatites: an inelastic neutron-scattering study. *Bone* **26**, 599-602 (2000).
23. C. Rey, J. L. Miquel, L. Facchini, A. P. Legrand, M. J. Glimcher, Hydroxyl groups in bone mineral. *Bone* **16**, 583-586 (1995).
24. C. Rey, C. Combes, C. Drouet, M. J. Glimcher, Bone mineral: update on chemical composition and structure. *Osteoporosis international* **20**, 1013-1021 (2009).
25. K. T. Lim, J. D. Suh, J. Kim, P. H. Choung, J. H. Chung, Calcium phosphate bioceramics fabricated from extracted human teeth for tooth tissue engineering. *Journal of Biomedical Materials Research Part B: Applied Biomaterials* **99**, 399-411 (2011).
26. M. Figueiredo *et al.*, Effect of the calcination temperature on the composition and microstructure of hydroxyapatite derived from human and animal bone. *Ceramics international* **36**, 2383-2393 (2010).
27. T. Sakae, H. Oinuma, M. Higa, Y. Kozawa, X-ray diffraction and FTIR study on heating effects of dentin from mammoth tusk. *Journal of Oral Biosciences* **47**, 83-88 (2005).
28. M. Albéric *et al.*, Relation between the macroscopic pattern of elephant ivory and its three-dimensional micro-tubular network. *PloS one* **12**, e0166671 (2017).
29. F. Berna *et al.*, Microstratigraphic evidence of in situ fire in the Acheulean strata of Wonderwerk Cave, Northern Cape province, South Africa. *Proceedings of the National Academy of Sciences* **109**, E1215-E1220 (2012).
30. E. Mendelovici, Comparative study of the effects of thermal and mechanical treatments on the structures of clay minerals. *Journal of Thermal Analysis and Calorimetry* **49**, 1385-1397 (1997).
